# Viral and Epidemiological Determinants of the Invasion Dynamics of Novel Dengue Genotypes

**DOI:** 10.1371/journal.pntd.0000894

**Published:** 2010-11-23

**Authors:** José Lourenço, Mario Recker

**Affiliations:** Department of Zoology, University of Oxford, Oxford, United Kingdom; Southwest Foundation for Biomedical Research (SFBR), United States of America

## Abstract

**Background:**

Dengue has become a major concern for international public health. Frequent epidemic outbreaks are believed to be driven by a complex interplay of immunological interactions between its four co-circulating serotypes and large fluctuations in mosquito densities. Viral lineage replacement events, caused for example by different levels of cross-protection or differences in viral fitness, have also been linked to a temporary change in dengue epidemiology. A major replacement event was recently described for South-East Asia where the Asian-1 genotype of dengue serotype 2 replaced the resident Asian/American type. Although this was proposed to be due to increased viral fitness in terms of enhanced human-to-mosquito transmission, no major change in dengue epidemiology could be observed.

**Methods/Results:**

Here we investigate the invasion dynamics of a novel, advantageous dengue genotype within a model system and determine the factors influencing the success and rate of fixation as well as their epidemiological consequences. We find that while viral fitness overall correlates with invasion success and competitive exclusion of the resident genotype, the epidemiological landscape plays a more significant role for successful emergence. Novel genotypes can thus face high risks of stochastic extinction despite their fitness advantage if they get introduced during episodes of high dengue prevalence, especially with respect to that particular serotype.

**Conclusion:**

The rarity of markers for positive selection has often been explained by strong purifying selection whereby the constraints imposed by dengue's two-host cycle are expected to result in a high rate of deleterious mutations. Our results demonstrate that even highly beneficial mutants are under severe threat of extinction, which would suggest that apart from purifying selection, stochastic effects and genetic drift beyond seasonal bottlenecks are equally important in shaping dengue's viral ecology and evolution.

## Author Summary

Dengue fever and the more severe dengue haemorrhagic fever and dengue shock syndrome are mosquito borne viral infections that have seen a major increase in terms of global distribution and total case numbers over the last few decades. There are currently four antigenically distinct and potentially co-circulating dengue serotypes and each serotype shows substantial genetic diversity, organised into phylogenetically distinct genotypes or lineages. While there is some evidence for positive selection, the evolutionary dynamics of dengue virus (DENV) is supposed to be mostly dominated by purifying selection due to the constraints imposed by its two-host life-cycle. Motivated by a recent genotype replacement event whereby the resident American/Asian lineage of dengue virus serotype 2 (DENV2) had been displaced by the fitter Asian-1 lineage we investigated some of the epidemiological factors that might determine the success and invasion dynamics of a novel, advantageous dengue genotype. Our results show that although small differences in viral fitness can explain the rapid expansion and fixation of novel genotypes, their fate is ultimately determined by the epidemiological landscape in which they arise.

## Introduction

Dengue virus (DENV) is the most wide-spread arbovirus affecting human populations. During the last decades it has increasingly become a major public health problem with significant economic and social impact [1]–[3]. It is transmitted between humans in urban and peri-urban settings predominantly by the *Aedes aegypti* and *Aedes albopictus* mosquitoes vector [4]. *Ae. aegypti* is extremely well adapted to urban environments where it efficiently breeds in artificial water containers, such as flower pots, plastic bags or discarded car tires, near human habitations. Both vectors have undergone rapid expansion worldwide in the last couple of decades leading to DENV endemicity in more than 100 countries [5].

There are four closely related and potentially co-circulating serotypes of DENV (DENV1-DENV4) [6], [7] and recovery from infection is believed to provide life-long immunity to the infecting serotype but only a brief period of heterologous protection to all other serotypes [8]. Most primary infections are self-limited and clinically silent but can occasionally result in a short-lived febrile illness which is commonly known as dengue fever (DF). In some cases this may progress to more severe and life-threatening illness such as dengue haemorrhagic fever (DHF) or dengue shock syndrome (DSS) [9]. While several risk factors for developing DHF/DSS have been described, including host genetic background, viral genotype, order of infecting serotype, time between infections or age of infection [1], [9], the most widely cited explanation is that of Antibody Dependent Enhancement (ADE) (e.g. [10]–[13]) whereby subneutralizing antibodies from primary infection can mediate viral entry into host cells leading to increased replication and disease manifestations [14]–[18].

**Figure 1:**
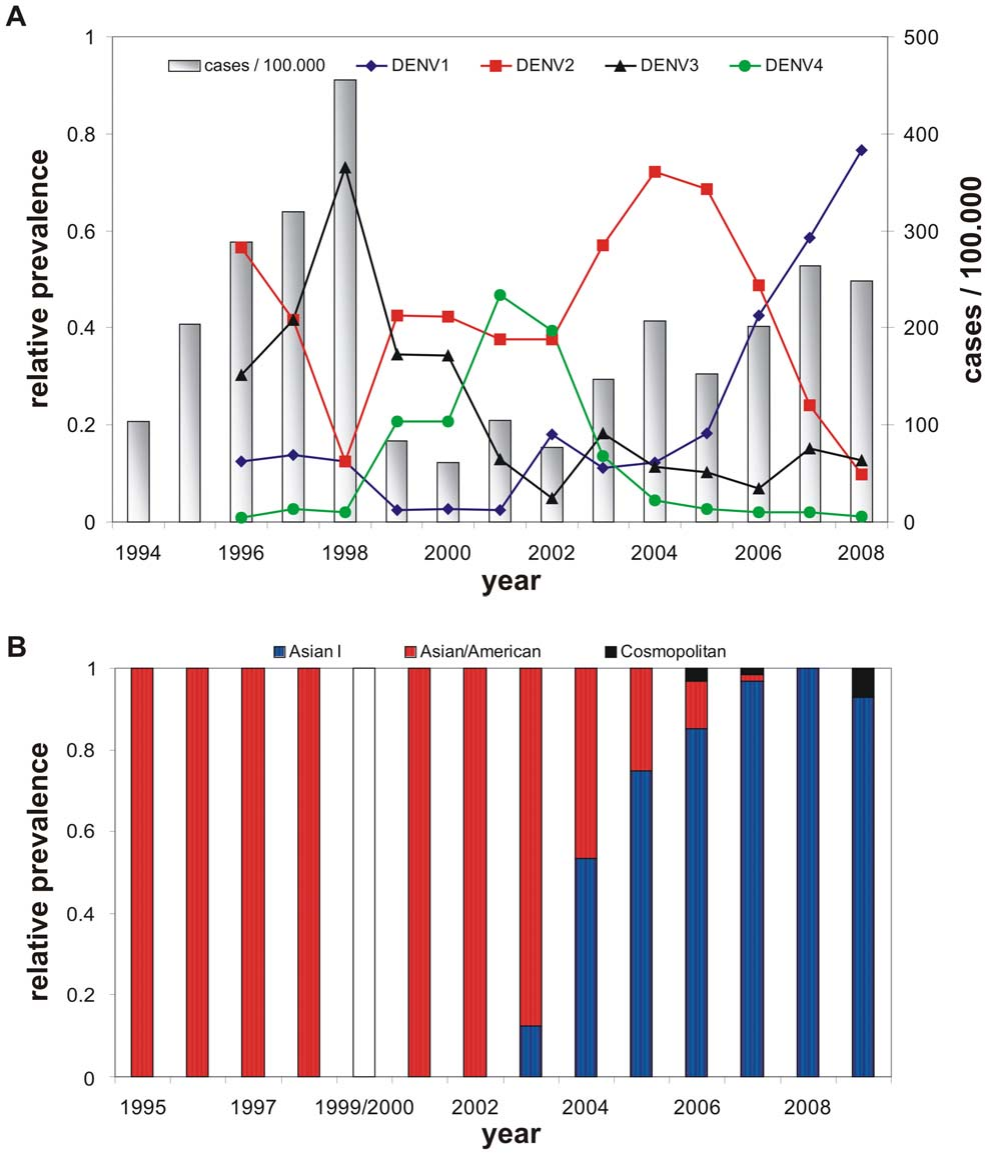
Dengue epidemiology in Southern Viet Nam. (A) The total number of hospitalised cases between 1994–2008 (bars) show the characteristic fluctuations in disease incidence with a big epidemic outbreak in 1998 followed by years of relatively low disease. The sequential replacement in dominance of one of dengue's four co-circulating serotypes (DENV1-DENV4) is clearly visible. (B) In the time between 2002 and 2008 Asian-1 genotype of serotype DENV2 (blue bars) competitively replaced the resident Asian/American type (red bars). Data for 1999 and 2000 missing; figure reproduced from Hang *et al.* [33].

The temporal epidemiological pattern of dengue is characterized by semi-periodic outbreaks whilst the inter-epidemic cycles in DF/DHF incidence highly correlate with the seasonal variations in vector population size (see e.g. [19]). Furthermore, individual serotype prevalences show cyclical replacements in dominance (Figure 1A) which are believed to be induced by the immune profile of the human population [20], [21].

Phylogenetic studies based on complete sequences of structural genes of all 4 serotypes have demonstrated the existence of multiple lineages in which different genotypes can be clustered [6], [7]. Despite a general bias in the literature towards studies based on single-gene approaches, spatio-temporal patterns of genotype replacement in endemic regions have been widely recovered from data [6], [7], [22]–[24]. With the extrinsic pressures on DENV, such as seasonal or human-forced reductions in vector population size or abundance and mobility of susceptible hosts, it has been proposed that genetic drift plays a major role in the observed phylodynamics [22], [25]. Furthermore, most studies have reported that DENV recent molecular evolution is marked by strong purifying selection, possibly due to the requirement of its two-host life cycle, and few reports have been able to show convincing evidence for positive selection either by the existence of non-synonymous mutations or in measures of fitness advantage in viral traits [6], [7], [23], [24], [26].

Following earlier reports of inter-serotypic difference in virulence (see e.g. [27]) one of the first convincing evidences for genetic determinants in disease outcome came from epidemiological studies suggesting that the DENV2 Asian genotype was associated with higher frequencies in DHF compared to the American genotype [28]. *In vitro* studies have since shown that the replication rate in both human monocyte-derived macrophages and dendritic cells as well as the vector's susceptibility were higher for the Asian genotype [29], [30]. It was also found that the Asian genotype of DENV2 had a slightly higher replication rate within the mosquito and a shorter extrinsic incubation period [31]. These results provided a rational explanation for the replacement patterns observed in the Americas, where displacement of the American genotype by the Asian genotype has taken place in several countries in recent years [28], [29], [32]. A similar lineage replacement event has also occurred in SE Asia, with Asian-1 lineage viruses having displaced Asian/American viruses from Viet Nam (Figure 1B), Cambodia and Thailand. This displacement was proposed to be due to difference in *in vivo* fitness, with higher viraemia levels observed in Asian-1 infected patients that could lead to an enhanced probability of human-to-mosquito transmission [33].

The study by Hang *et al.* [33] demonstrated some other intriguing aspects about the invasion dynamics of Asian-1. A phylogenetic analysis suggested that the Asian genotype was introduced into the population years before it had been detected, and once it was detected it reached fixation within a relatively short period of time. The rate at which this genotype replaced the Asian/American type would suggest a significant fitness advantage not only over the resident genotype but possibly also over the other circulating serotypes; however, there was no discernible difference in the overall epidemiological dynamics in the period before or after fixation. Although these results suggested that a fitness advantage in a specific viral trait played a decisive role, the emergence of advantageous genotypes are as likely to be driven by the level of transmission and the underlying immune status of the human population.

Here we have constructed an epidemiological model of dengue to qualitatively address the impact of immunity and transmission on the invasion and replacement patterns of a novel advantageous dengue genotype. Our results suggest that the observed replacement events can be explained by competition between genotypes of relatively small fitness differences which, although sufficient for displacement, do not interfere with the overall serotype dynamics. Furthermore, we show that invasion success and total time required for fixation are strongly influenced by inter- and intra-serotype competition at the time of introduction.

## Methods

### Description of the model

The model is an extension of the 4-serotype mathematical framework analysed by Recker et al. [34] and includes a mosquito vector component, temporary cross-immunity after primary infection and seasonal forcing in mosquito biting. In summary, we disregard the effect of maternal antibodies and instead assume that human individuals are born susceptible to all 4 serotypes. After recovery from primary infection they acquire life-long immunity to the infecting serotype and cross-immunity to any other serotype for a short period of time. As temporary immunity wanes, individuals become susceptible to secondary heterologous infection. For simplicity and because of the relative rarity of reported third and fourth infections we assume that after recovery from secondary infections individuals remain fully protected against further challenges [4], [35]. The system can then be given by the following set of differential equations describing the rate of change in humans either susceptible, infected with, temporarily immune or recovered from dengue serotypes 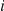, 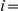
DENV1, DENV2, DENV2′, DENV3 or DENV4:
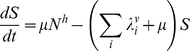
(1)

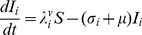
(2)

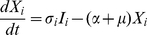
(3)

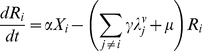
(4)

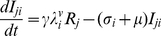
(5)

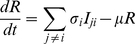
(6)with the force of infection of serotype 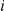 affecting the human population, 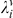, given as
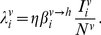
(7)We denote 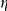 as the mosquito biting rate and 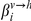 as the vector-to-human transmission probability; 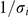 and 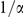 are the respective durations of infection and cross-immunity. Given the short period of infection we do not account for the possibility of co-infections by two or more serotypes. We assume a constant human population size 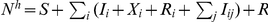 and further assume that infection has a negligible effect on the average death rate, 
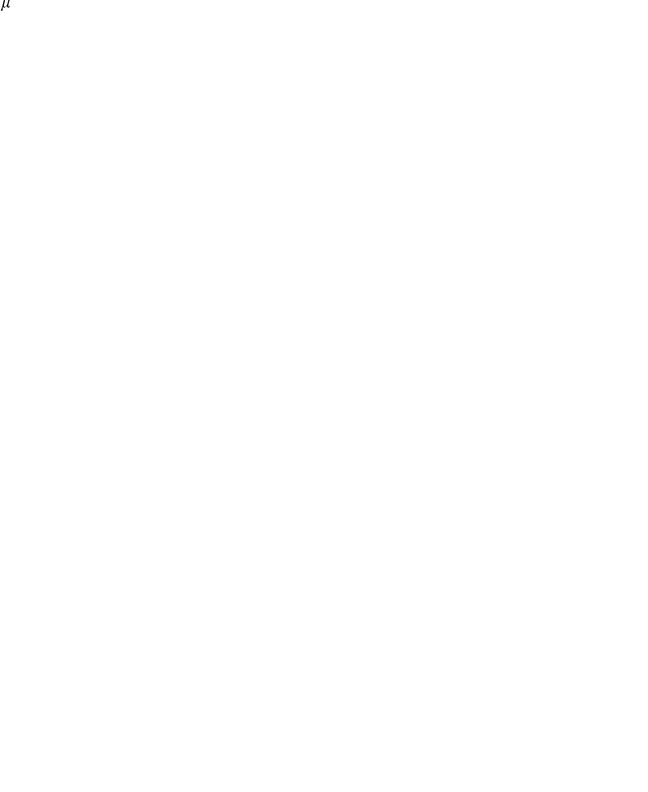
. To account for seasonal variation we assume a periodically forced biting rate, that is we set
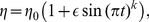
(8)where 
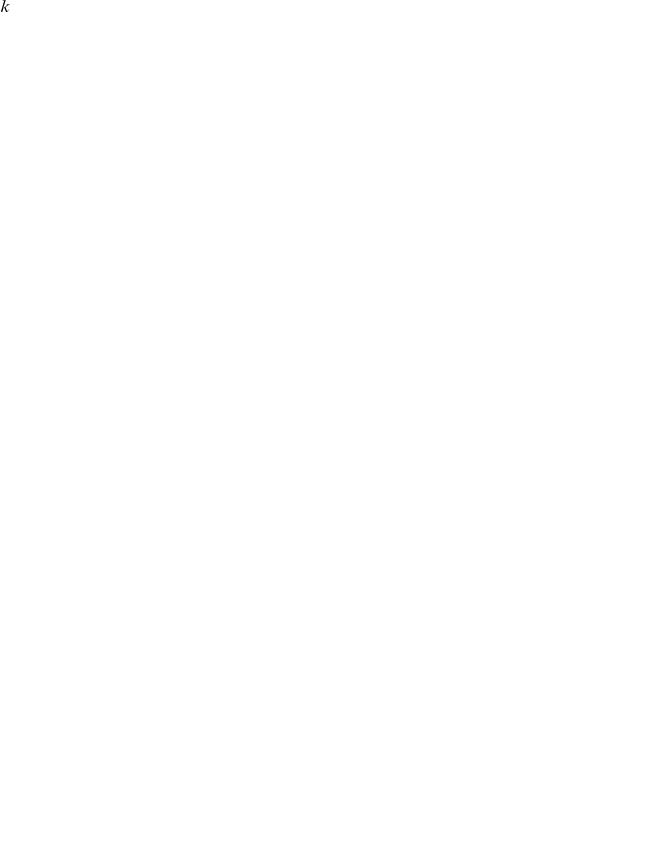
 is a positive integer influencing the ‘seasonality’ where 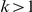 results in shorter and more pronounced seasons.

The dynamics of the mosquito population is given as follows:
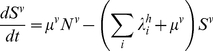
(9)

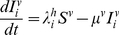
(10)with the force of infection from humans to mosquitoes given as
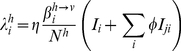
(11)


In accordance with our previous model [34] we assume that antibody-dependent enhancement acts to increase both susceptibility to and transmissibility of secondary heterologous infection by factors 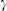 and 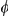, respectively, with values described in Table 1.

**Table 1:** Model Parameters.

parameter	definition	value
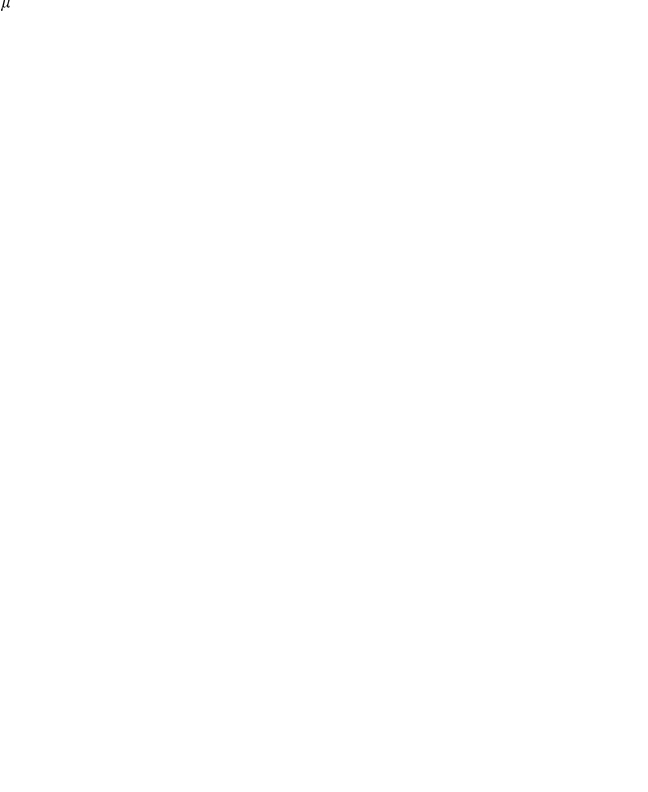	host lifespan	70 years
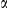	temporary heterologous immunity	5 months
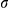	infectious period	3.65 days
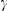	susceptibility enhancement	1.33
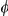	transmissibility enhancement	1.66
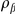	increase in probability of human-to-mosquito transmission	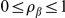
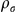	increase in infectious period	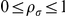
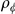	increase in enhancement of secondary infections	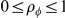
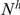	host population size	9 million
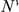	vectore population size	22.5 million
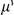	vector lifespan	16 days
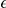	amplitude in seasonality	0.3
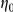	biting rate	115 per year
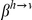	transmission probability human 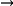 mosquito	0.9
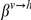	transmission probability mosquito 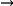 human	0.8
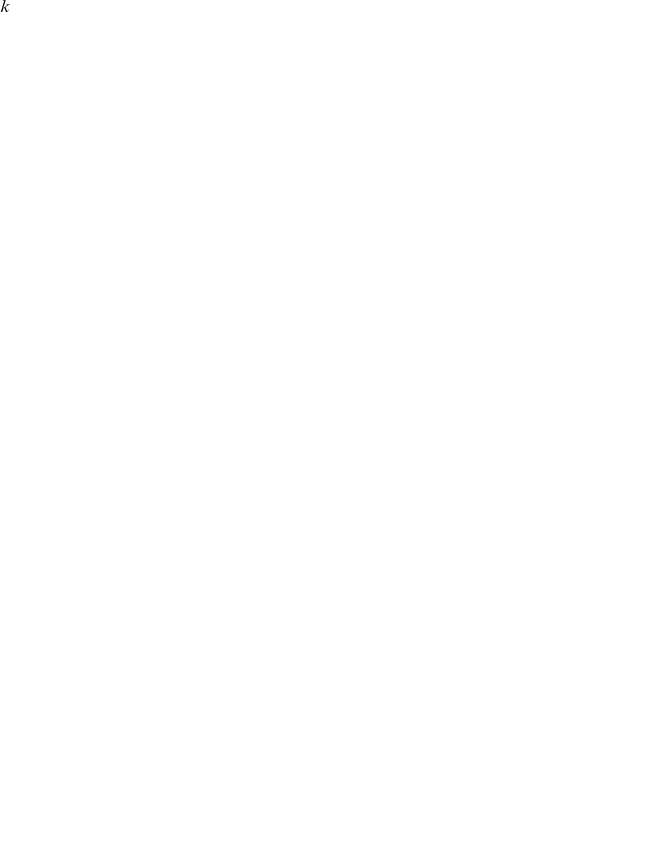	speed in seasonality change	2
	detection threshold (relative frequency)	10%
	deterministic fixation threshold (relative frequency)	99%
	deterministic number of introduced DENV2′ (cases)	1 infected mosquito
	stochastic fixation threshold (cases)	0
	stochastic number of introduced DENV2′ (cases)	2 per infectious class

Parameter values used in the deterministic and stochastic simulations.

To investigate the invasion patterns of a novel and fitter dengue genotype we assume that DENV2 is represented by two genotypes which differ in relative fitness but are antigenically equivalent. That is, individuals previously infected by DENV2 are immune to type DENV2′ and vice versa. We consider four different fitness traits which we can vary independently: (i) transmissibility from human to mosquito, e.g. through increased viral load, 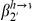, (ii) longer life-expectancy of mosquitoes infected with DENV2′ to emulate a shorter extrinsic incubation period (EIP), 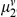, (iii) longer infectious period in humans, 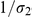, and (iv) an increased level of enhancement of secondary infections, 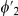. These can simply be given using:
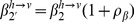
(12)

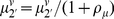
(13)

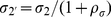
(14)

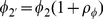
(15)That is, 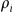, can be considered as the degree of the fitness advantage. In line with the suggestion by Hang *et al.* [33], most of our analysis is concentrated on the fitness advantage due to increased viral load and thus transmissibility from the infected human individual to the mosquito vector, 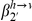. In fact, we found that the results presented here are invariant to the actual viral trait that is enhanced; results obtained under changes to other viral traits can be found in the supporting material.

### Stochastic simulations

To address certain aspects of the invasion process of a more probabilistic nature, such as invasion success rates and fixation events, we also implemented the above model as a stochastic framework using a tau-leap Gillespie algorithm [36]. Stochastic simulations were initialized with equilibrium population status derived from the deterministic framework with parameter values the same as given in Table 1 (see Figure S7 and S8 for general model behaviour).

## Results

We used a simple epidemiological model of dengue to investigate the effect of host population immunity structures and transmission settings on the invasion pattern of a novel DENV2 genotype, hereby denoted as DENV2′. The model is based on a previously introduced deterministic, multi-serotype framework (e.g. [34], [37], [38]) but extended to include the mosquito vector population, with seasonal fluctuations in biting frequencies, and a period of temporary cross-immunity; full model details are given in the Methods section. We verified our model predictions within a stochastic framework which allowed us to more adequately address and further explore certain aspects of the invasion and replacement dynamics and their determinants [39].

**Figure 2:**
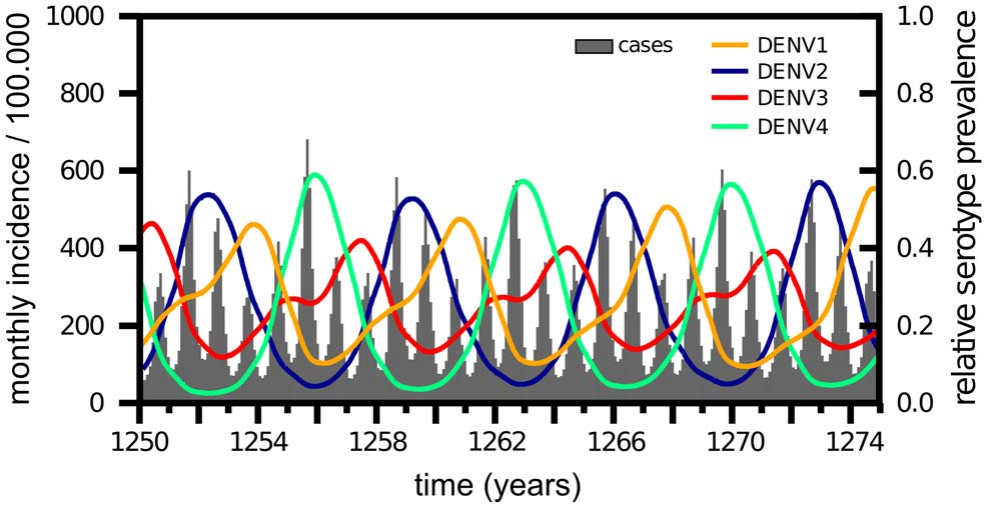
General model behaviour. Under parameter values given in Table 1 the model reproduces the typical epidemiological pattern of dengue, showing the cyclical behaviour in serotype prevalence (coloured lines) and semi-regular epidemic outbreaks (total incidence per month, grey line).

The general dynamics generated by our model under parameter values given in Table 1 and prior to the introduction of a novel DENV2 genotype are characterised by semi-regular epidemic outbreaks and asynchronous cyclical behaviour in serotype prevalence (Figure 2). In accordance with previous studies (e.g. [34], [37], [40]) a wide range of incidence and serotype dynamics with different inter-epidemic periods can also be found under changes to key parameters values, especially those relating to the degree of enhancement of secondary infection or the period of temporary cross-immunity (Figures S1 and S2). For the remainder of this work, however, we kept most parameter values constant to allow for better comparisons between invasion patterns and their epidemiological determinants.

### Genotype invasion and replacement

**Figure 3:**
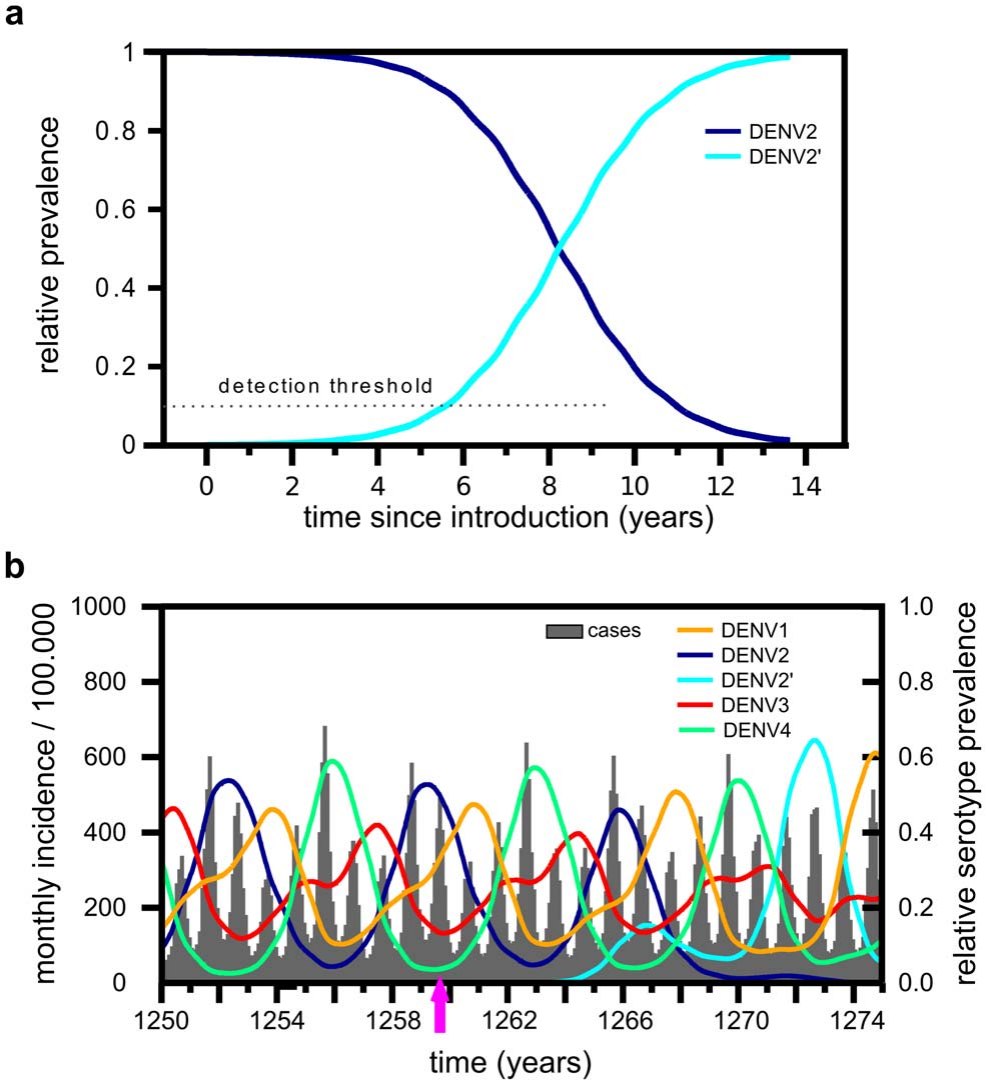
Dynamics of an invading genotype. (A) Plotting the frequency of DENV2′ relative to DENV2 highlights two phases of the invasion process: a period of very low frequency and a subsequent rapid shift in dominance and competitive exclusion. The fitness advantage in both plots is due to increased human-to-vector transmission rate 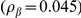 over the resident type. (B) The cyclical serotype behaviour remains invariant to the introduction of a fitter genotype of serotype 2, DENV2′ (cyan line), which enters the population at time 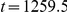 (pink arrow) and drives the resident type, DENV2 (blue line), to extinction after 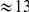 years. Comparing the equivalent time series in Figure 2, no major changes in disease levels or inter-epidemic period can be observed. Other parameters as in Table 1.

We examined the dynamics of a novel genotype introduced into a dengue endemic population by either an infected human individual or via an infected mosquito. The novel genotype is here denoted as DENV2′, to represent the Asian-1 genotype of serotype 2, whereas the resident type is denoted as DENV2 to represent the Asian/American type. Figure 3 shows the result of an invasion scenario where the invading genotype has a small fitness advantage over the resident type (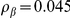, corresponding to a fitness advantage of 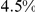). In this case, higher viral fitness was realised through enhanced transmissibility from infected human individuals to the mosquito vectors, i.e. 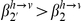. In agreement with the data, two important features of the invasion dynamics can be observed and are highlighted in Figure 3B. Despite the eventual fast rate at which the advantageous genotype replaces the resident type, there is a significant lag between the point of introduction and the time when DENV2′ genotype would reach a detectable level of prevalence within the population; we refer to this level of prevalence as detection threshold. Furthermore, despite the expected temporary rise in dengue incidence, compared to the situation without invasion, the overall dynamics in both disease incidence and serotype prevalence remain largely invariant (Figure 3A). This suggests that both the time lag between introduction and first detection and also the rapid exclusion of the resident genotype, such as reported by Hang *et al.* [33], can be explained by a relatively small fitness advantage of the invading genotype.

The same qualitative behaviour can be also found when changing other viral traits which could determine the fitness advantage. That is, shortening the extrinsic incubation period, 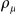, increasing the duration of infection, 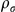, or the level of enhancement of secondary infection, 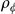, have the same effect as increasing the transmission probability from infected humans to mosquitoes, 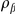. Notably, though, when considering low advantages, smaller differences in terms of viral fitness are required to achieve the same rate of fixation if the fitness advantage manifests itself in longer infectious periods compared to an increase in transmissibility (Figure S3). Interestingly, while similar levels of fitness advantages in either EIP or transmissibility result in the same fixation times (Figure S4), the disturbance on the epidemiological pattern of dengue is less severe when the fitness advantage is expressed in the mosquito (Figure S5). From now on, we concentrate only on a fitness advantage through the proposed increase in human-to-vector transmission.

### The effect of viral fitness and time of introduction

**Figure 4:**
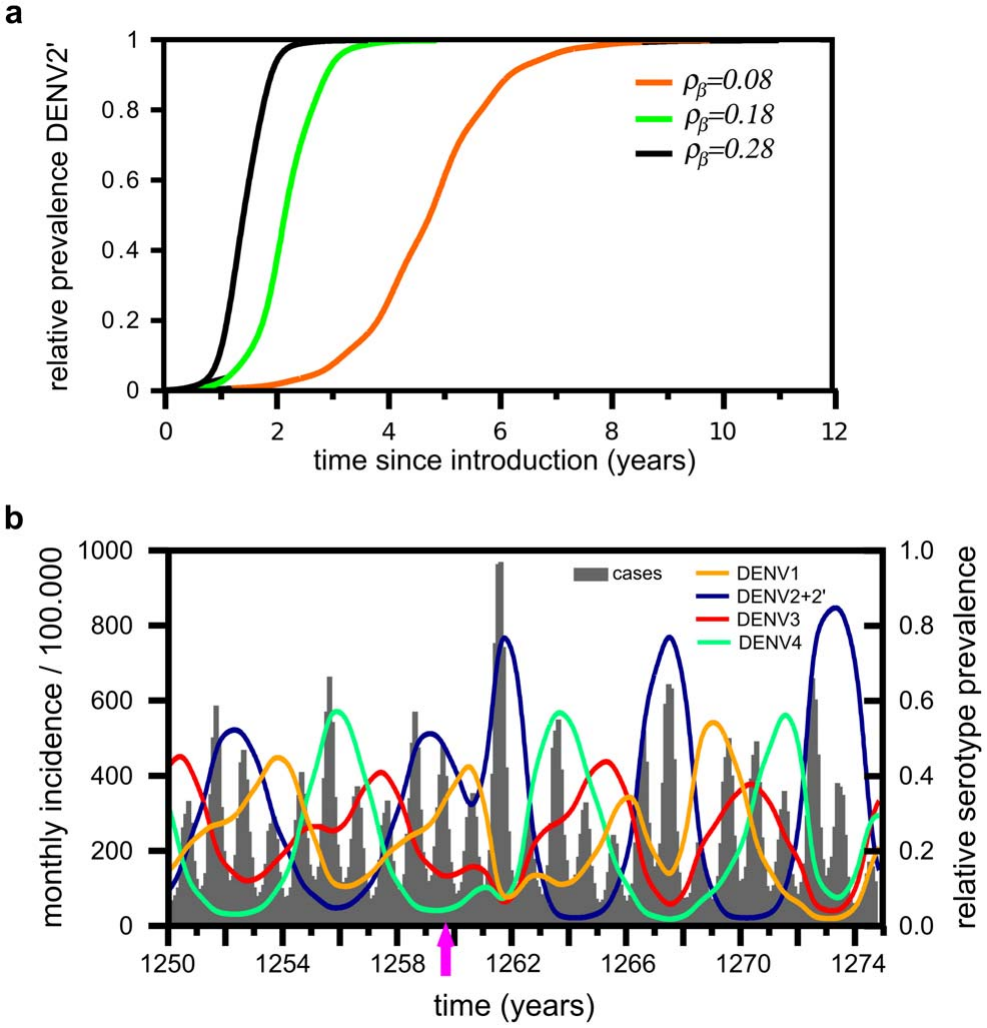
The effect of viral fitness on fixation time and epidemiological patterns. (A) The graph demonstrates the increased rate in competitive exclusion of the resident genotype, DENV2, for increasing levels of viral fitness of the invading type, DENV2′, with 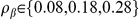. Higher fitness advantages significantly reduce the period of low level prevalence and the overall time to fixation. (B) Higher fitness advantages, here 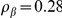, can have a significant effect on both incidence and serotype dynamics, causing a big epidemic outbreak followed by a severe trough in serotype 2 frequency. Other parameter values as in Table 1.

As shown in Figure 3, a small increase in transmissibility from human to mosquito seems sufficient for a novel genotype to displace a resident type within a short period of time. The actual rate of competitive exclusion and overall time from introduction of the advantageous genotype to its fixation in the population is likely to depend on various factors including fitness advantage, rate of transmission and immune profile within the human population. As shown in Figure 4A, increasing viral fitness accelerates the rate at which the invading genotype drives the resident type, DENV2, to extinction, resulting in a shorter period between introduction and fixation. For example, increasing the fitness advantage from 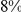 to 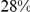 reduces the time to fixation from 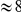 years down to 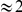 years. However, this increase in viral fitness has a major effect on dengue incidence patterns and the dynamics of the other serotypes. In this case it leads to a significantly bigger epidemic outbreak at the time of replacement followed by a long period of low transmission and low prevalence of serotype 2 which could endanger its continuous persistence; this is highlighted in Figure 4B (compare to Figure 3A).

**Figure 5:**
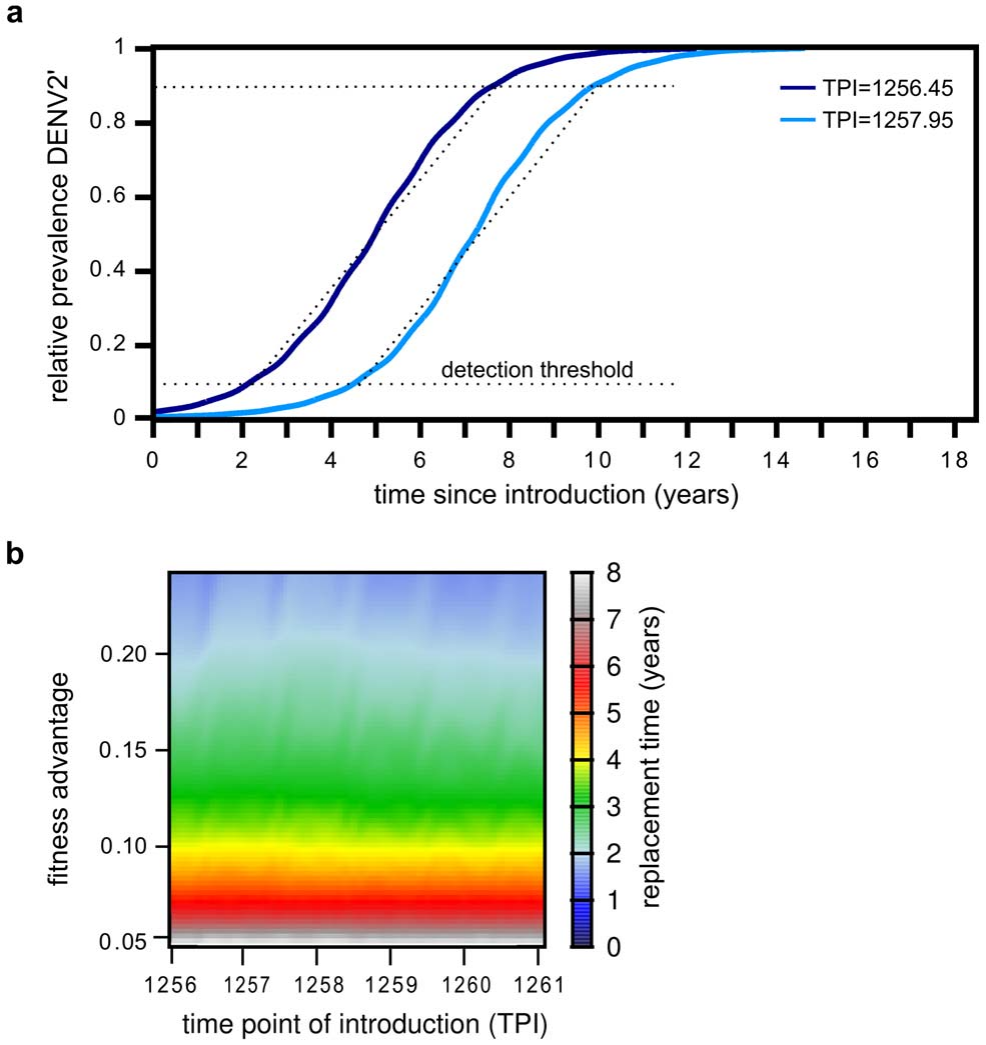
The effect of the time of introduction on the rate of fixation. (A) The graph shows the increase in the frequency of DENV2′, relative to DENV2, for two different time points of introduction (TPI). Despite a discernible difference in the total time for DENV2′ to reach fixation and competitively exclude the resident type, the actual rate of displacement (highlighted as dashed lines) remains the same. That is, the differences in fixation times in both cases are solely due to the differences in the initial expansion period of the invading genotype before it reaches wide-spread detection level (here arbitrarily set at 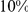 relative prevalence). (B) Whereas the relative fitness advantage of the invading genotype has a significant effect on the rate of replacement, it remains invariant to the time at which it is introduced into the population. All parameters as in Table 1 and 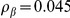 for (A).

We next addressed the effect of the time of introduction on the invasion dynamics. This was simply motivated by the fact that serotype competition is not constant over time but is strongly affected by the level of transmission which itself is dependent on host immunity level and seasonal variation in mosquito densities. Not surprisingly, we found that the time of introduction can significantly alter the time taken for a novel genotype to reach fixation. Figure 5A shows the decrease in the frequency of DENV2, relative to the fitter genotype DENV2′, for two different time points of introduction. However, while the overall duration from invasion to fixation is dependent on the time when DENV2′ gets introduced, the actual rate of replacement remains constant. In other words, the time taken from DENV2′ passing a detection threshold, relative to DENV2, to reaching fixation is independent of the time of introduction (Figure 5B) and therefore independent of the overall epidemiological dynamics. This, on the other hand, suggests that the time lag between introduction and the point when it has spread sufficiently for detection, or waiting time, is strongly influenced by the epidemiological profile at that time.

To investigate further the determinants for fixation time we simulated a number of invasion events at various time points over a four year period and recorded the total time to fixation for each event with respect to (i) the number of naive individuals, (ii) serotype 2 susceptible individuals, (iii) disease prevalence and (iv) mosquito biting frequency. While we could not find a clear correlation between any of these population profiles and fixation time, we observed a trend for longer fixation times during the time window where the relative prevalence of serotype 2 was increasing (Figure S6).

### The effect of serotype competition on emergence time and invasion success

**Figure 6:**
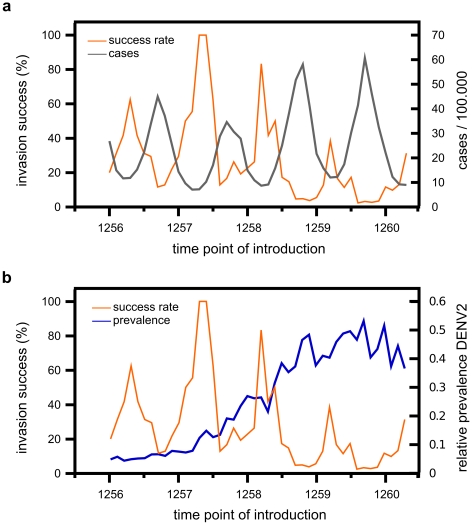
The effect of transmission and serotype competition on invasion success. The success rate of the invading genotype, DENV2′, strongly varies depending on the number of total infected individuals and the relative prevalence of serotype 2 in the population at the time point of introduction (TPI). (A) The invasion success (orange line) oscillates out of phase with total dengue incidence (grey line) and is minimized when disease prevalence peaks, demonstrating how the current level of transmission can influence the invasion success of new advantageous genotypes. (B) The highest rates of successful invasions can be observed during periods of low relative prevalence of serotype 2 (blue line). In contrast, the probability of an invading advantageous genotype to get established and reach fixation is significantly reduced as serotype 2 gains wide-spread dominant within the population. Parameters as in Table 1 and 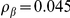.

The results from our deterministic model suggest that novel genotypes can face long periods at very low prevalence before breaching a detection threshold and going to fixation. Within a more realistic setting these periods signify an enhanced risk of stochastic extinction of the novel type despite its fitness advantage over the resident type. To better address the invasion success of DENV2′ we used a stochastic formulation of our model (see Methods) and simulated a number of invasion events over a period of four years and recorded the success rate of invasion, here defined as the successful introduction into a population followed by competitive exclusion of the resident type. As demonstrated in Figure 6A we observed that invasion success shows an oscillatory behaviour whose phase seems negatively correlated to total dengue prevalence at time of introduction. This suggests that the invasion of a newly advantageous genotype can be hampered by serotype competition during epidemics and favoured during off-season periods. Moreover, the amplitude of oscillation, i.e. the maximum success rate, is dependent on and again negatively correlated to serotype 2 prevalence. Figure 6B shows the increase in relative prevalence of DENV2 over the 4-year period which clearly correlates with a decline in the success rate of DENV2′.

**Figure 7:**
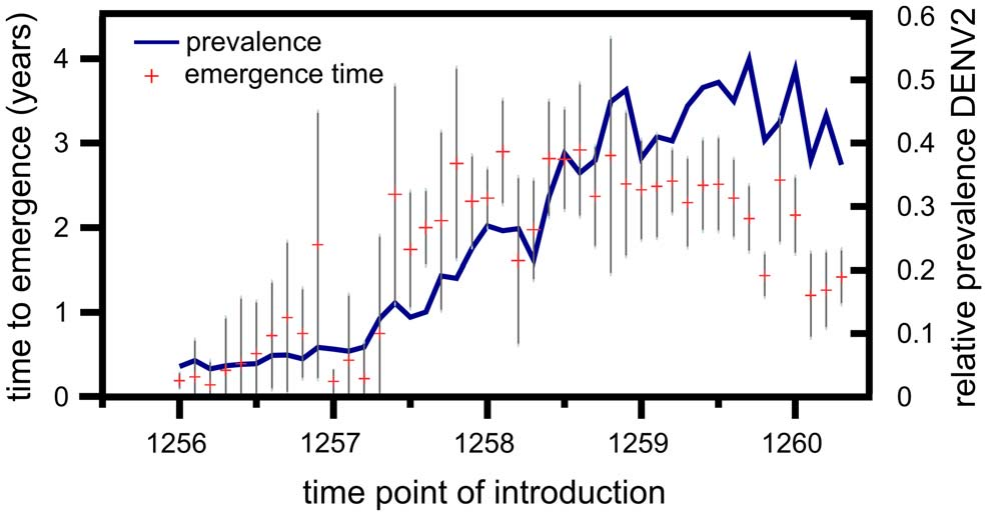
The effect of serotype competition on the emergence time of successful fixation events. The total time required for a novel (and eventually successful) genotype DENV2′ to reach detection level is highly dependent on the relative prevalence of serotype 2 at the time it enters the population. The red crosses show how the average emergence times, i.e. the period between introduction and reaching a 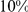 detection threshold, of successful invasion events increases with the relative prevalence of DENV2 at the time of introduction (blue line). Standard deviations, based on 10 simulated successful invasion events, are shown as grey bars. Parameters as in Table 1 and 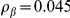.

Since the time taken from passing a detection threshold to reaching fixation was shown to be independent of the time of introduction (Figure 5B), we focused on the relationship between serotype 2 prevalence and the time to emergence, i.e. the period between introduction and reaching a 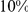 prevalence threshold. Figure 7 clearly illustrates that a novel and advantageous genotype entering the population during periods of high DENV2 prevalence will face significantly longer emergence times than those introduced during periods of low prevalence. Together our results indicate that the fate of a novel genotype is strongly determined by both inter- and intra-serotype competition at the time of introduction.

## Discussion

We analysed the invasion pattern of a novel dengue genotype into an endemic population with 4 co-circulating serotypes. Within our framework we assumed that the invading genotype, representing the Asian-1 genotype of dengue virus serotype 2, possesses a fitness advantage over the resident type, the Asian/American genotype, through enhanced transmissibility from infected human individuals to the mosquito vectors. This assumption was based on the findings by Hang *et al.* [33] which showed increased plasma viraemia levels in patients infected by Asian-1 DENV2 viruses. In contrast to other studies [30], [41], Hang and colleagues did not find increased infectivity of Asian-1 viruses to *Ae. aegypti* mosquitoes *per se*; however, it is easy to envisage how higher viral titers could enhance the ‘per bite’ probability of human-to-vector transmission. By thus focusing on the hypothesis of a small increase in transmissibility during primary and secondary infections, and in agreement with the data, we observed that the total time for genotype replacement is composed of a period during which the invading type can circulate at very low prevalence levels for several years, followed by a rapid shift in dominance and competitive exclusion after the invading genotype had emerged; here we defined ‘emergence’ as a threshold level of prevalence where widespread detection would be highly likely.

Of particular interest is the time lag between introduction and emergence, or waiting time, when the detection of the new dengue genotype might be difficult by surveillance systems based on low viral sampling numbers and/or infrequent genotyping. Not surprisingly, we found that this period is strongly and positively affected by the difference in viral fitness between the resident and novel genotype. In the case of small fitness advantages several years could pass before the invading type has spread sufficiently to outcompete the resident type on a population-wide level. Furthermore, as the epidemiological pattern would remain largely invariant, passive surveillance systems based simply on case numbers could also easily fail to detect this intra-serotype replacement event. These results therefore support the findings of Hang *et al.* [33] who hypothesised that a small enhancement of human-to-mosquito transmission through increased viral load is sufficient to explain the observed invasion pattern in Southern Viet Nam where Asian-1 was first detected in 2003 despite the phylogenetic analyses dating the introductory event sometime during the late 1990's.

Apart from increased transmission from infected humans to the mosquito vectors we also considered other viral traits that could be enhanced in the Asian-1 genotype, such as longer infectious periods or shorter extrinsic incubation periods (EIP). The latter is of particular interest as it can potentially lead to a significantly increase in vectorial capacity [31]. While the actual viral trait which is enhanced does not alter the overall invasion pattern or results presented in this work (Figures S3, S4, S5, S9, S10, and S11), we found that viral fitness traits have an additive effect (Figure S4). This means that even smaller individual enhancements are sufficient to explain the observed invasion dynamics of the Asian-1 genotype, especially under the assumption that this replacement event did not have a major effect on the sero-epidemiological pattern of dengue. Interestingly, though, our results suggest that dengue incidence and serotype dynamics are less disturbed when the fitness advantage is manifested through shorter EIP than increased infectivity or transmissibility (Figure S5).

In addition to viral fitness, the time point at which a novel genotype enters a population is crucially important in determining its invasion dynamics and ultimately success. Whereas the relative fitness advantage affects the overall time between introduction and fixation, the epidemiological profile more strongly determines the period of low level prevalence before the advantageous genotype emerges. We tested various epidemiological factors for their influence on the waiting time but to our surprise only found the relative prevalence of DENV2 to have a strong effect. That is, whereas population susceptibility to either dengue in general or serotype 2 in particular had no immediate influence on the time between introduction and wide-spread detection, we found that the relative prevalence of DENV2 at the time of introduction positively correlates with extended periods during which the novel genotype circulates below a detection threshold. Therefore, while transmission intensities strongly affect the success of an invasion event, the dominance level of serotype 2 within the population determines both the invasion success rate and, independently, the period before the invading genotype would reach a sufficient level of prevalence to be widely detecable. Our results thus confirm that serotype interactions and the resulting epidemiological landscape can have a big influence on intra-serotype dynamics and thus viral evolution, as previously noted by Zhang and colleagues [23].

There is considerable interest in determining the evolutionary processes that underlie the observed structures and genetic variation of dengue virus populations (both inter- and intra-serotypic). Overall, low estimates of selection pressure, in terms of average 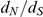 values, and the fact that dengue has a two-host life-cycle are commonly used to place purifying selection as the strongest selective force acting on dengue evolution [23], [26], [42]. However, it is also clear that dengue viruses exhibit strong spatio-temporal variations. Various phylogenetic studies have identified frequent DENV lineage turnover events which have resulted in the characteristic, ladder-like tree (e.g. [24], [42]) and which are commonly ascribed to positive selection [24], [32], [43]. In addition, genetic drift has also been proposed to play a major part in dengue evolution such that the replacement of viral lineages or clades could be explained through stochastic processes alone. For example, repeated bottlenecks due to large seasonal fluctuations in mosquito densities imply that the emergence of novel and possibly advantageous genotypes could be a recurrent phenomenon followed by a strong probability for extinction in the subsequent circulating seasons which could explain the weak signature for positive selection in the data (compared to purifying selection). This in turn would also suggest that the success of a genotype does not always reflect its viral fitness [7]. In fact, we have shown that novel genotypes, especially those that arise during large epidemic outbreaks, can face high risks of extinction despite possessing a fitness advantage. Furthermore, even successful genotypes, i.e. those that eventually reach fixation, potentially undergo prolonged periods of low frequency which can span for several transmission seasons independently of the epidemics therein. Therefore, low measures of adaptive selection in this case would not necessarily imply strong purifying selection but could equally be explained by other epidemiological factors. This, however, needs to be confirmed within a more rigorous framework.

Dengue's two-host life-cycle implies a significant evolutionary constraint whereby the majority of newly arising variants are likely to be deleterious and selectively removed from the population. We have shown that even novel and advantageous DENV genotypes can undergo periods of several years prior reaching sufficiently large population sizes to escape the risk of extinction. Our results thus indicate that in addition to purifying selection, the epidemiological landscape and stochastic effects might be equally important determinants in shaping the viral evolutionary ecology.

## Supporting Information

Figure S1 **Model behaviour under different levels of enhancement.** Under a wide range of parameter values, the model reproduces the observed epidemiological pattern of dengue. In agreement with previous models, the level of ADE, either in terms of transmission or susceptibility enhancement (ϕ and γ, respectively), has a significant effect on the qualitative dynamics, with greater degrees of ADE generally leading to more pronounced epidemic outbreak and chaotic serotype oscillations. These simulated time series show the cyclical behaviour in serotype prevalence (coloured lines) and regular epidemic outbreaks (grey) for (A) ϕ = γ = 1.0 (B) ϕ = γ = 1.3 (C) ϕ = 1.9 γ = 1.3 (D) ϕ = 1.3 γ = 1.9. Other parameter values as in Table 1 (main text).
(1.57 MB TIF)

Figure S2 **Model behaviour under different levels of temporary heterologous immunity.** Under various periods of temporary heterologous immunity (α), the model reproduces the observed epidemiological pattern of dengue. Increasing the value of α - (A) 3.5, (B) 4.5, (C) 5.5, (D) 6.5 - leads to higher interepidemic periods as epidemics caused by one serotype build temporary immunity and prevent DENV from exploring the human population until immunity wanes.
(1.60 MB TIF)

Figure S3 **The effect of viral fitness assuming changes in infectious period and secondary infections.** The graph demonstrates the increased rate in competitive exclusion of the resident genotype DENV2 for increasing levels of viral fitness of DENV2′ expressed as (A) infectious period (ρ_σ_) and (B) increased infectivity in secondary infections (ρ_Φ_). (A) Similar fitness differences are required for displacement to take place in the same time window as in Figure 4, main text. (B) Higher fitness differences are required for displacement to take place in the same time window as in Figure 4, main text. Other parameter values as in Table 1 (main text).
(0.45 MB TIF)

Figure S4 **The synergistic effect of viral fitness assuming changes in the extrinsic incubation period and human-to-vector transmission.** The graph demonstrates the increased rate in competitive exclusion of the resident genotype DENV2 for increasing levels of viral fitness of DENV2′ expressed as a shorter extrinsic incubation period (ρμ) and increased human-to-vector transmission (ρ_β_) (see Methods in main text). (A,B) Equal fitness differences either expressed as shorter extrinsic incubation period or increased human-to-vector transmission lead to similar emergence and fixation times. (C) The effect of ρ_μ_ and ρ_β_ on the invasion dynamics is additive. Other parameter values as in Table 1 (main text).
(1.19 MB TIF)

Figure S5 **The effect of viral fitness assuming changes extrinsic incubation period.** The graph demonstrates the increased rate in competitive exclusion of the resident genotype DENV2 for increasing levels of viral fitness of DENV2′ expressed as a shorter extrinsic incubation period (ρ_μ_) (see Methods). (A) Higher fitness differences lead to shorter waiting and fixation times. (B) Interestingly, even significant advantages, here ρ_μ_ = 0.2, i.e. a 20% fitter genotype, does not result in severe disruption of the incidence patterns of dengue. Other parameter values as in Table 1 (main text).
(1.21 MB TIF)

Figure S6 **Effects of other population status on total time of fixation.** The graphs show the time taken for a novel serotype 2 genotype to reach fixation given (A) the number of susceptible (naïve) individuals, (B) dengue disease prevalence, (C) number of susceptible individuals to serotype 2 and (D) seasonality, at the time point of introduction of the invading genotype (black curves). Points represent an introduction event, given a certain population status, and are coloured according to the total time for fixation. A clear increase in total time is observed in all 4 plots along the chosen time window with no correlation between any of the variables in A,B,C or D. ρ_β_ = 0.045 all other parameter values as in Table 1 (main text).
(1.16 MB TIF)

Figure S7 **Stochastic model behaviour.** Initialized with the population state and parameters of the deterministic model at t = 1250, the stochastic model exhibits a similar time series as presented in Figure 2 (main text) with persistence of all serotypes. This simulated time series show the cyclical behaviour in serotype prevalence (coloured lines) and regular epidemic outbreaks (grey). Parameter values as in Table 1 (main text).
(0.96 MB TIF)

Figure S8 **Effect of fitness advantage on invasion success.** Considering a fixed time point for introduction, increasing values of ρ_β_ result in higher invasion success rates of DENV2′ and lowers fixation time. Time of introduction 1259.5, parameter values as in Table 1 (main text).
(0.29 MB TIF)

Figure S9 **The effect of transmission and serotype competition on invasion success and emergence time of successful fixation events, assuming changes in the EIP.** The success rate of the invading genotype, DENV2′, strongly varies depending on the number of total infected individuals and the relative prevalence of serotype 2 in the population at the time point of introduction (TPI). The total time required for a novel (and eventually successful) genotype DENV2′ to reach detection level is highly dependent on the relative prevalence of serotype 2 at the time it enters the population. (A) The invasion success (orange line) oscillates out of phase with total dengue incidence (grey line) and is minimized when disease prevalence peaks, demonstrating how the current level of transmission can influence the invasion success of new advantageous genotypes. (B) The highest rates of successful invasions can be observed during periods of low relative prevalence of serotype 2 (blue line). In contrast, the probability of an invading advantageous genotype to get established and reach fixation is significantly reduced as serotype 2 gains wide-spread dominant within the population. (C) The red points show how the average emergence times, i.e. the period between introduction and reaching a 10% detection threshold, of successful invasion events increases with the relative prevalence of DENV2 at the time of introduction (blue line). Standard deviations, based on 10 simulated successful invasion events, are shown as light-blue bars. Parameters as in Table 1 and ρ_μ_ = 0.045 for S9.
(1.22 MB TIF)

Figure S10 **The effect of transmission and serotype competition on invasion success and emergence time of successful fixation events, assuming changes in human infectious period.** The success rate of the invading genotype, DENV2′, strongly varies depending on the number of total infected individuals and the relative prevalence of serotype 2 in the population at the time point of introduction (TPI). The total time required for a novel (and eventually successful) genotype DENV2′ to reach detection level is highly dependent on the relative prevalence of serotype 2 at the time it enters the population. (A) The invasion success (orange line) oscillates out of phase with total dengue incidence (grey line) and is minimized when disease prevalence peaks, demonstrating how the current level of transmission can influence the invasion success of new advantageous genotypes. (B) The highest rates of successful invasions can be observed during periods of low relative prevalence of serotype 2 (blue line). In contrast, the probability of an invading advantageous genotype to get established and reach fixation is significantly reduced as serotype 2 gains wide-spread dominant within the population. (C) The red points show how the average emergence times, i.e. the period between introduction and reaching a 10% detection threshold, of successful invasion events increases with the relative prevalence of DENV2 at the time of introduction (blue line). Standard deviations, based on 10 simulated successful invasion events, are shown as light-blue bars. Parameters as in Table 1 and ρ_σ_ = 0.045.
(1.23 MB TIF)

Figure S11 **The effect of transmission and serotype competition on invasion success and emergence time of successful fixation events, assuming changes in transmissibility of secondary infections.** The success rate of the invading genotype, DENV2′, strongly varies depending on the number of total infected individuals and the relative prevalence of serotype 2 in the population at the time point of introduction (TPI). The total time required for a novel (and eventually successful) genotype DENV2′ to reach detection level is highly dependent on the relative prevalence of serotype 2 at the time it enters the population. (A) The invasion success (orange line) oscillates out of phase with total dengue incidence (grey line) and is minimized when disease prevalence peaks, demonstrating how the current level of transmission can influence the invasion success of new advantageous genotypes. (B) The highest rates of successful invasions can be observed during periods of low relative prevalence of serotype 2 (blue line). In contrast, the probability of an invading advantageous genotype to get established and reach fixation is significantly reduced as serotype 2 gains wide-spread dominant within the population. (C) The red points show how the average emergence times, i.e. the period between introduction and reaching a 10% detection threshold, of successful invasion events increases with the relative prevalence of DENV2 at the time of introduction (blue line). Standard deviations, based on 10 simulated successful invasion events, are shown as light-blue bars. Parameters as in Table 1 and ρ_Φ_ = 0.075.
(1.20 MB TIF)
