# Supplementary figures and images for: Viral and Epidemiological Determinants of the Invasion Dynamics of Novel Dengue Genotypes
Source: PLoS Negl Trop Dis. 2010 Nov 23;4(11):e894. doi: 10.1371/journal.pntd.0000894 (PMC2990689; doi:10.1371/journal.pntd.0000894)

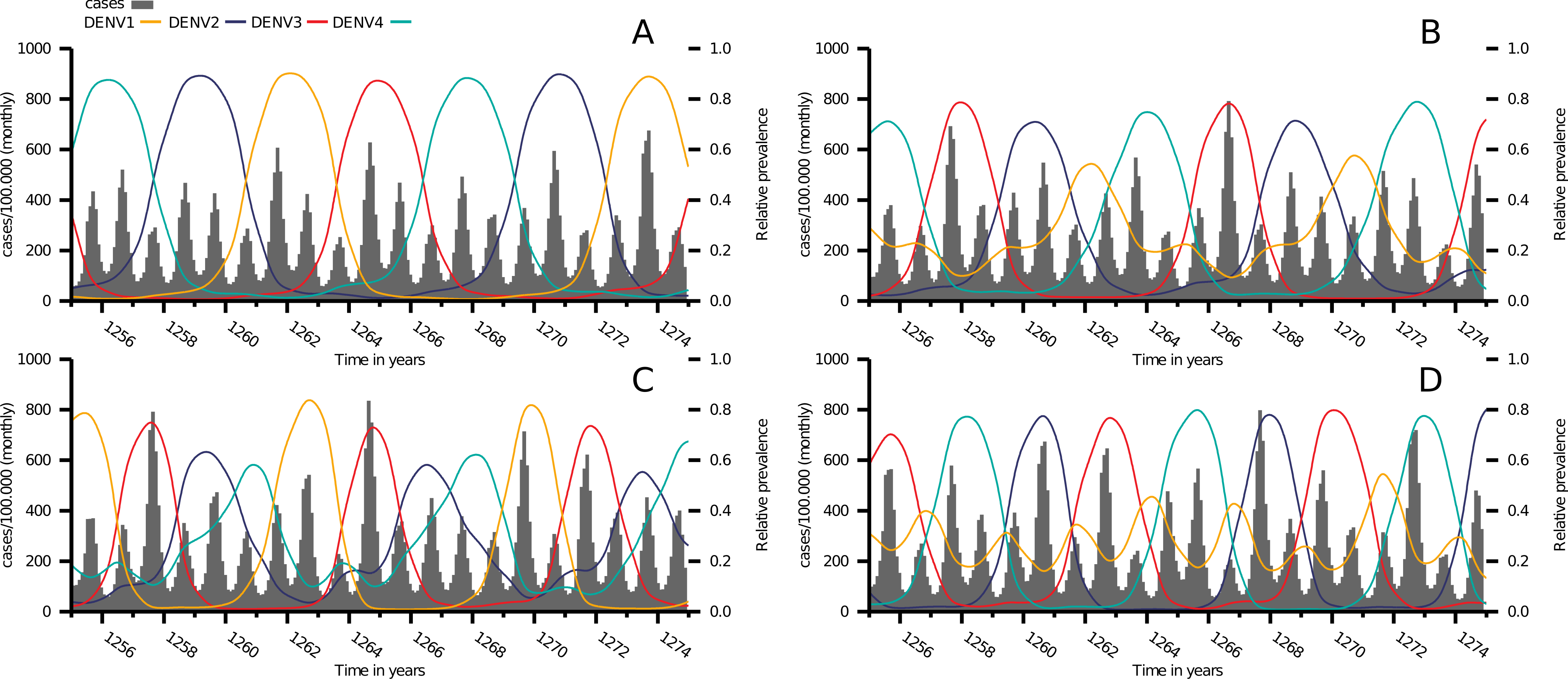

Supplement: Figure S1 — Model behaviour under different levels of enhancement. Under a wide range of parameter values, the model reproduces the observed epidemiological pattern of dengue. In agreement with previous models, the level of ADE, either in terms of transmission or susceptibility enhancement (ϕ and γ, respectively), has a significant effect on the qualitative dynamics, with greater degrees of ADE generally leading to more pronounced epidemic outbreak and chaotic serotype oscillations. These simulated time series show the cyclical behaviour in serotype prevalence (coloured lines) and regular epidemic outbreaks (grey) for (A) ϕ = γ = 1.0 (B) ϕ = γ = 1.3 (C) ϕ = 1.9 γ = 1.3 (D) ϕ = 1.3 γ = 1.9. Other parameter values as in Table 1 (main text). (1.57 MB TIF) [file pntd.0000894.s001.tif]

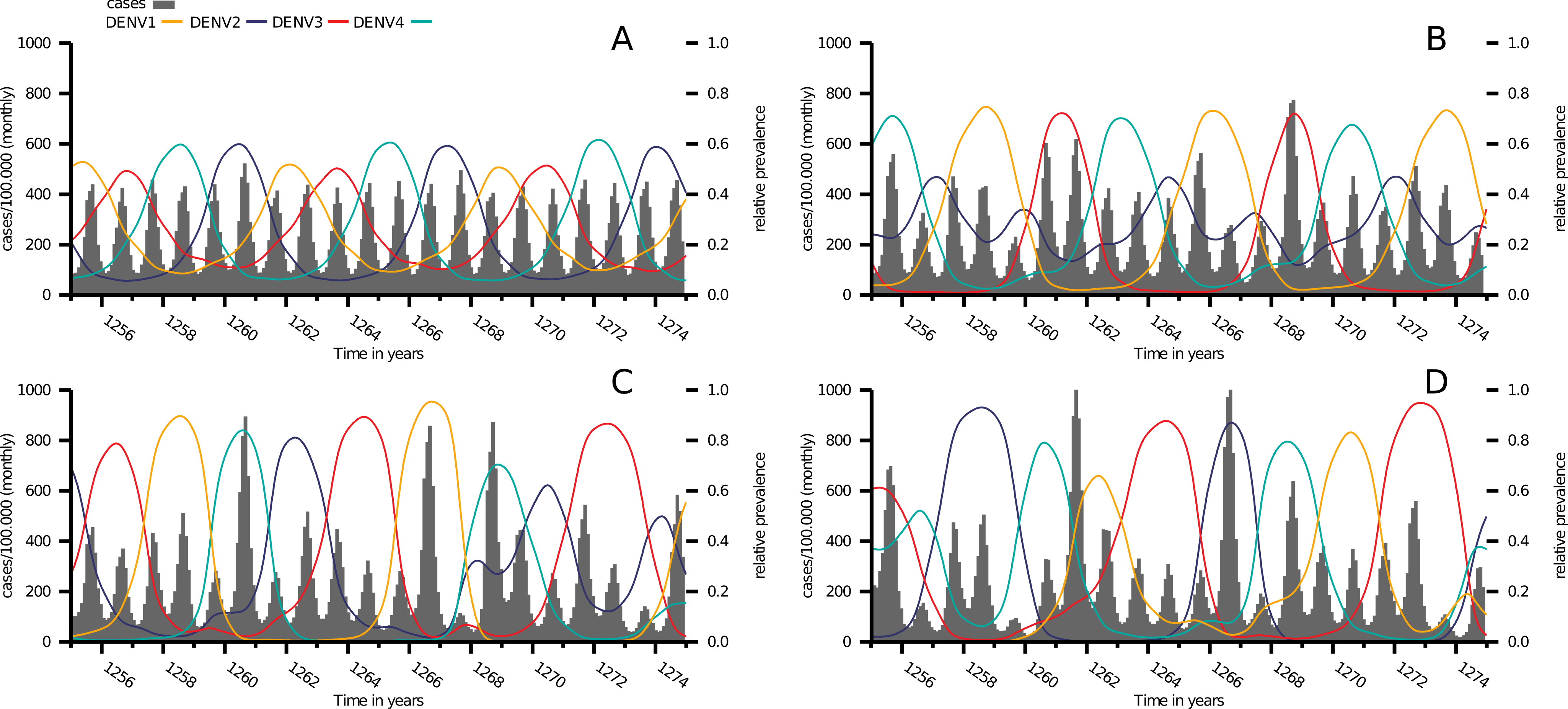

Supplement: Figure S2 — Model behaviour under different levels of temporary heterologous immunity. Under various periods of temporary heterologous immunity (α), the model reproduces the observed epidemiological pattern of dengue. Increasing the value of α - (A) 3.5, (B) 4.5, (C) 5.5, (D) 6.5 - leads to higher interepidemic periods as epidemics caused by one serotype build temporary immunity and prevent DENV from exploring the human population until immunity wanes. (1.60 MB TIF) [file pntd.0000894.s002.tif]

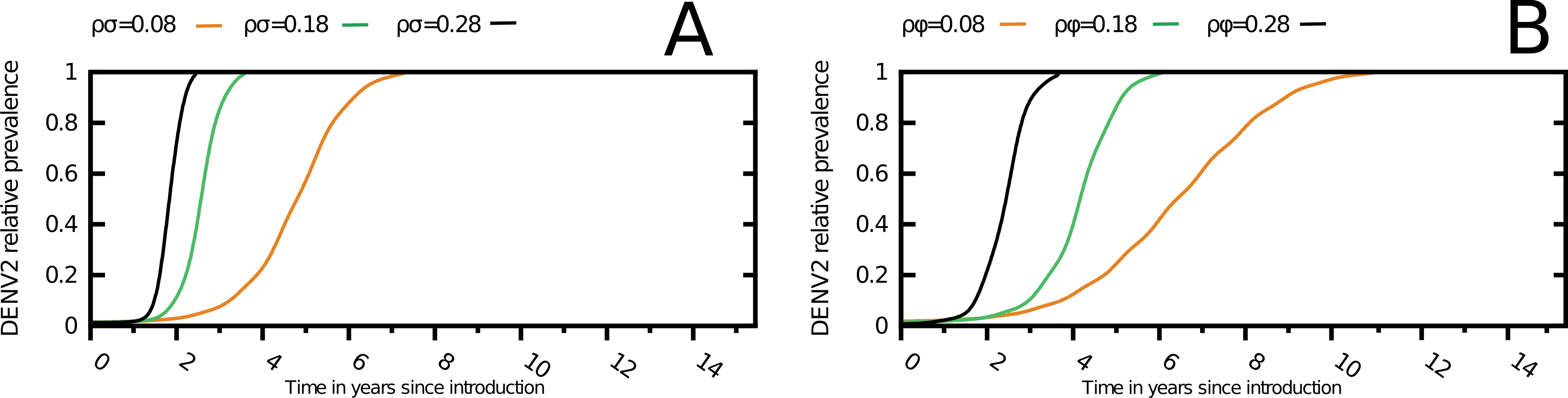

Supplement: Figure S3 — The effect of viral fitness assuming changes in infectious period and secondary infections. The graph demonstrates the increased rate in competitive exclusion of the resident genotype DENV2 for increasing levels of viral fitness of DENV2′ expressed as (A) infectious period (ρσ) and (B) increased infectivity in secondary infections (ρΦ). (A) Similar fitness differences are required for displacement to take place in the same time window as in Figure 4, main text. (B) Higher fitness differences are required for displacement to take place in the same time window as in Figure 4, main text. Other parameter values as in Table 1 (main text). (0.45 MB TIF) [file pntd.0000894.s003.tif]

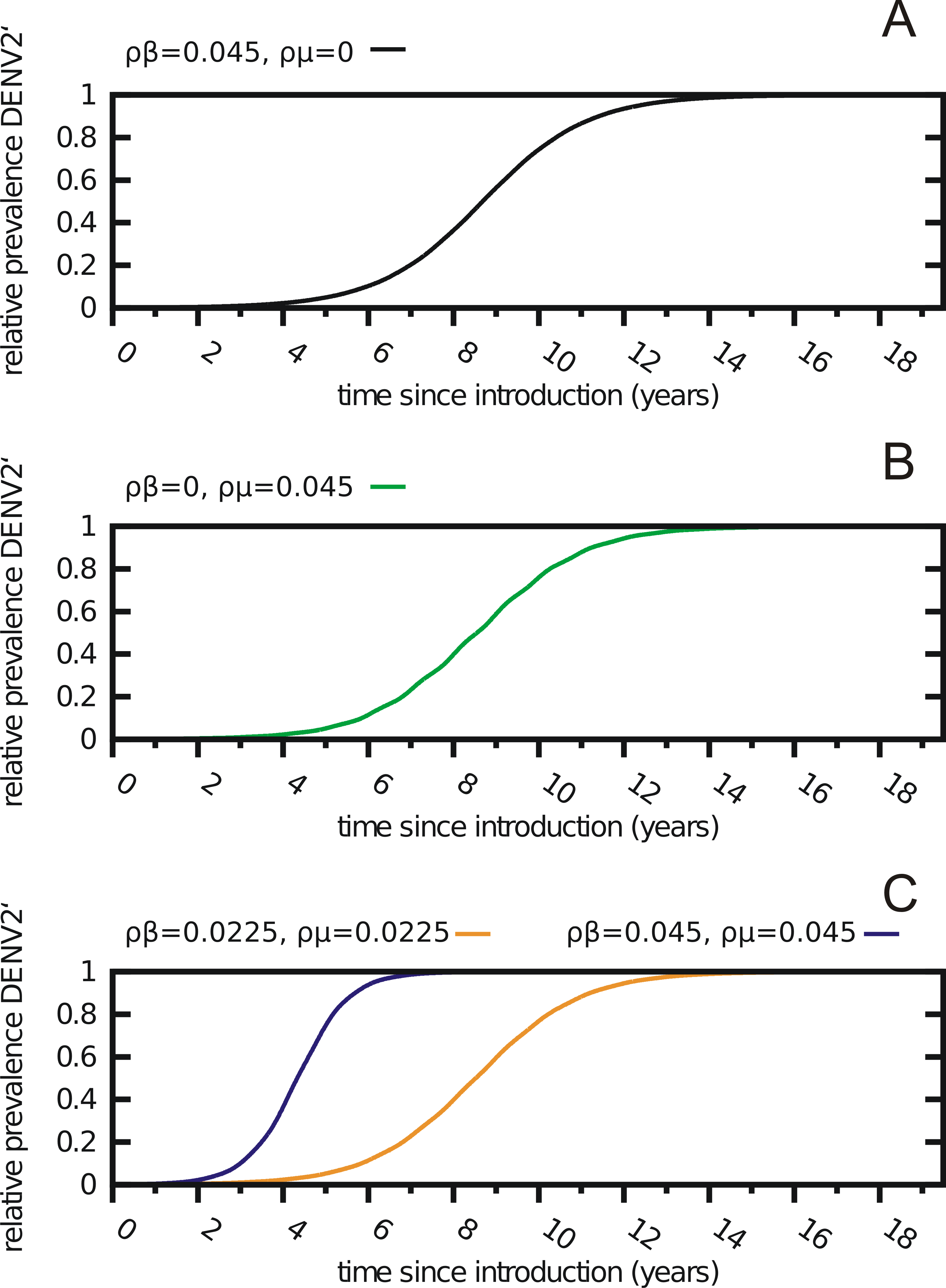

Supplement: Figure S4 — The synergistic effect of viral fitness assuming changes in the extrinsic incubation period and human-to-vector transmission. The graph demonstrates the increased rate in competitive exclusion of the resident genotype DENV2 for increasing levels of viral fitness of DENV2′ expressed as a shorter extrinsic incubation period (ρμ) and increased human-to-vector transmission (ρβ) (see Methods in main text). (A,B) Equal fitness differences either expressed as shorter extrinsic incubation period or increased human-to-vector transmission lead to similar emergence and fixation times. (C) The effect of ρμ and ρβ on the invasion dynamics is additive. Other parameter values as in Table 1 (main text). (1.19 MB TIF) [file pntd.0000894.s004.tif]

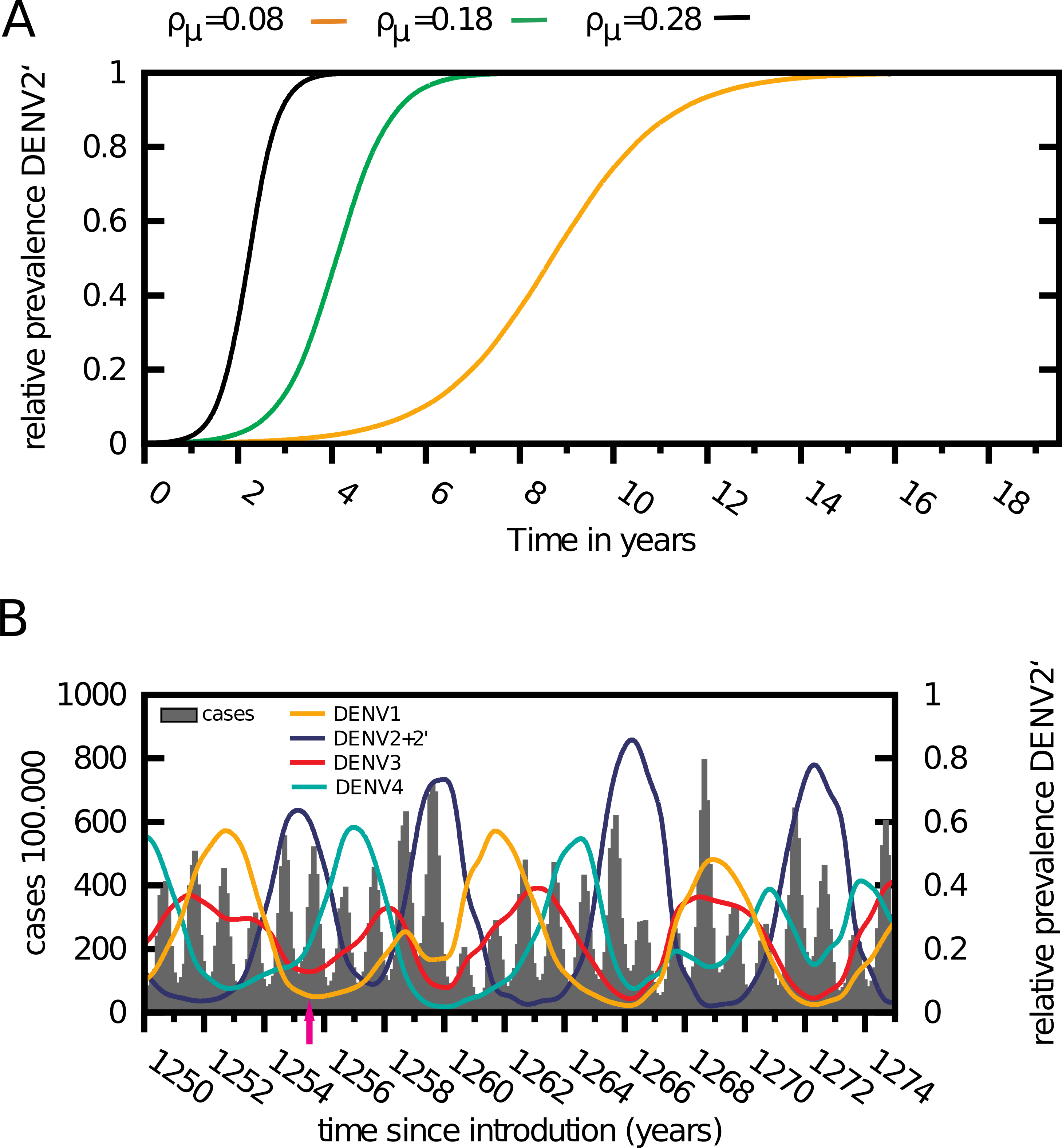

Supplement: Figure S5 — The effect of viral fitness assuming changes extrinsic incubation period. The graph demonstrates the increased rate in competitive exclusion of the resident genotype DENV2 for increasing levels of viral fitness of DENV2′ expressed as a shorter extrinsic incubation period (ρμ) (see Methods). (A) Higher fitness differences lead to shorter waiting and fixation times. (B) Interestingly, even significant advantages, here ρμ = 0.2, i.e. a 20% fitter genotype, does not result in severe disruption of the incidence patterns of dengue. Other parameter values as in Table 1 (main text). (1.21 MB TIF) [file pntd.0000894.s005.tif]

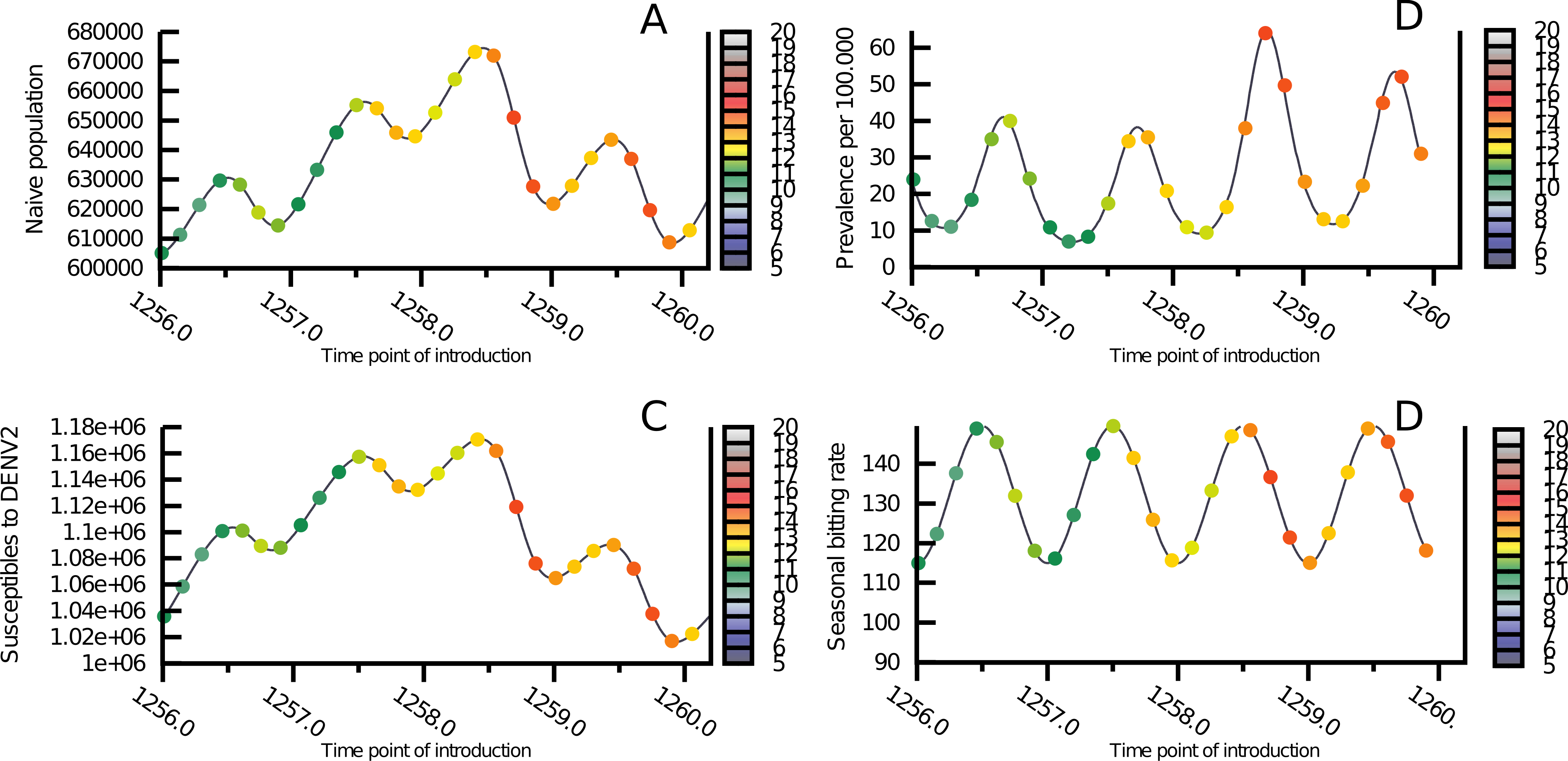

Supplement: Figure S6 — Effects of other population status on total time of fixation. The graphs show the time taken for a novel serotype 2 genotype to reach fixation given (A) the number of susceptible (naïve) individuals, (B) dengue disease prevalence, (C) number of susceptible individuals to serotype 2 and (D) seasonality, at the time point of introduction of the invading genotype (black curves). Points represent an introduction event, given a certain population status, and are coloured according to the total time for fixation. A clear increase in total time is observed in all 4 plots along the chosen time window with no correlation between any of the variables in A,B,C or D. ρβ = 0.045 all other parameter values as in Table 1 (main text). (1.16 MB TIF) [file pntd.0000894.s006.tif]

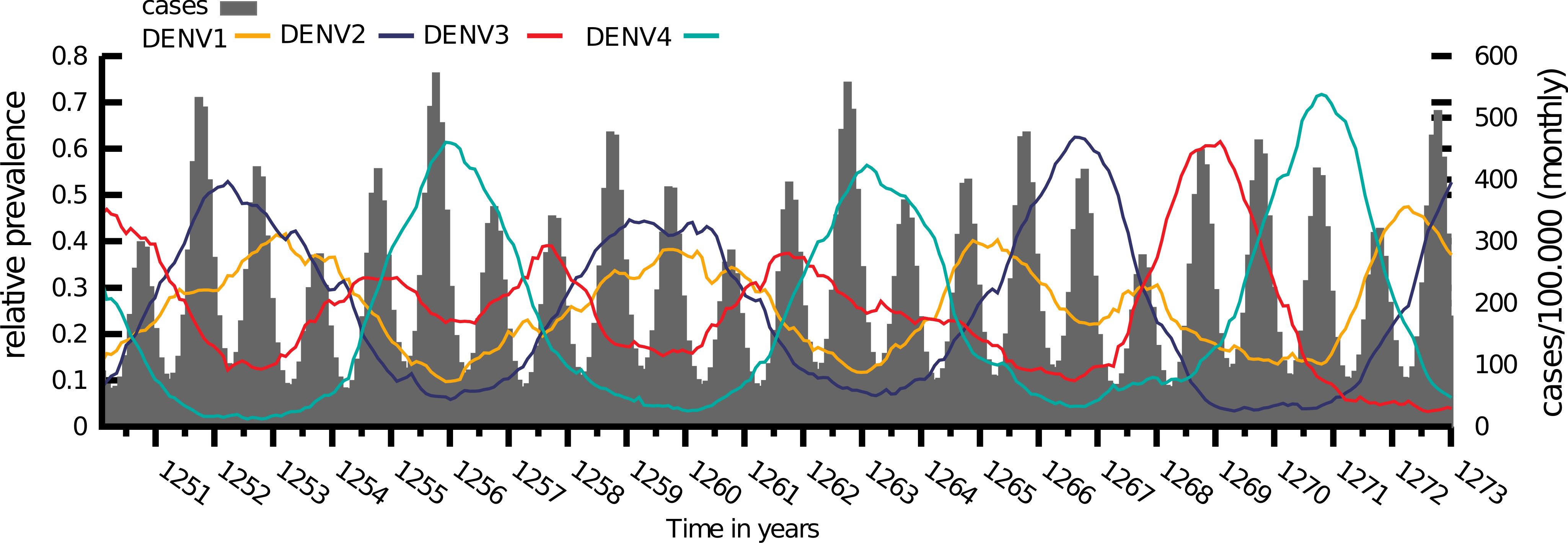

Supplement: Figure S7 — Stochastic model behaviour. Initialized with the population state and parameters of the deterministic model at t = 1250, the stochastic model exhibits a similar time series as presented in Figure 2 (main text) with persistence of all serotypes. This simulated time series show the cyclical behaviour in serotype prevalence (coloured lines) and regular epidemic outbreaks (grey). Parameter values as in Table 1 (main text). (0.96 MB TIF) [file pntd.0000894.s007.tif]

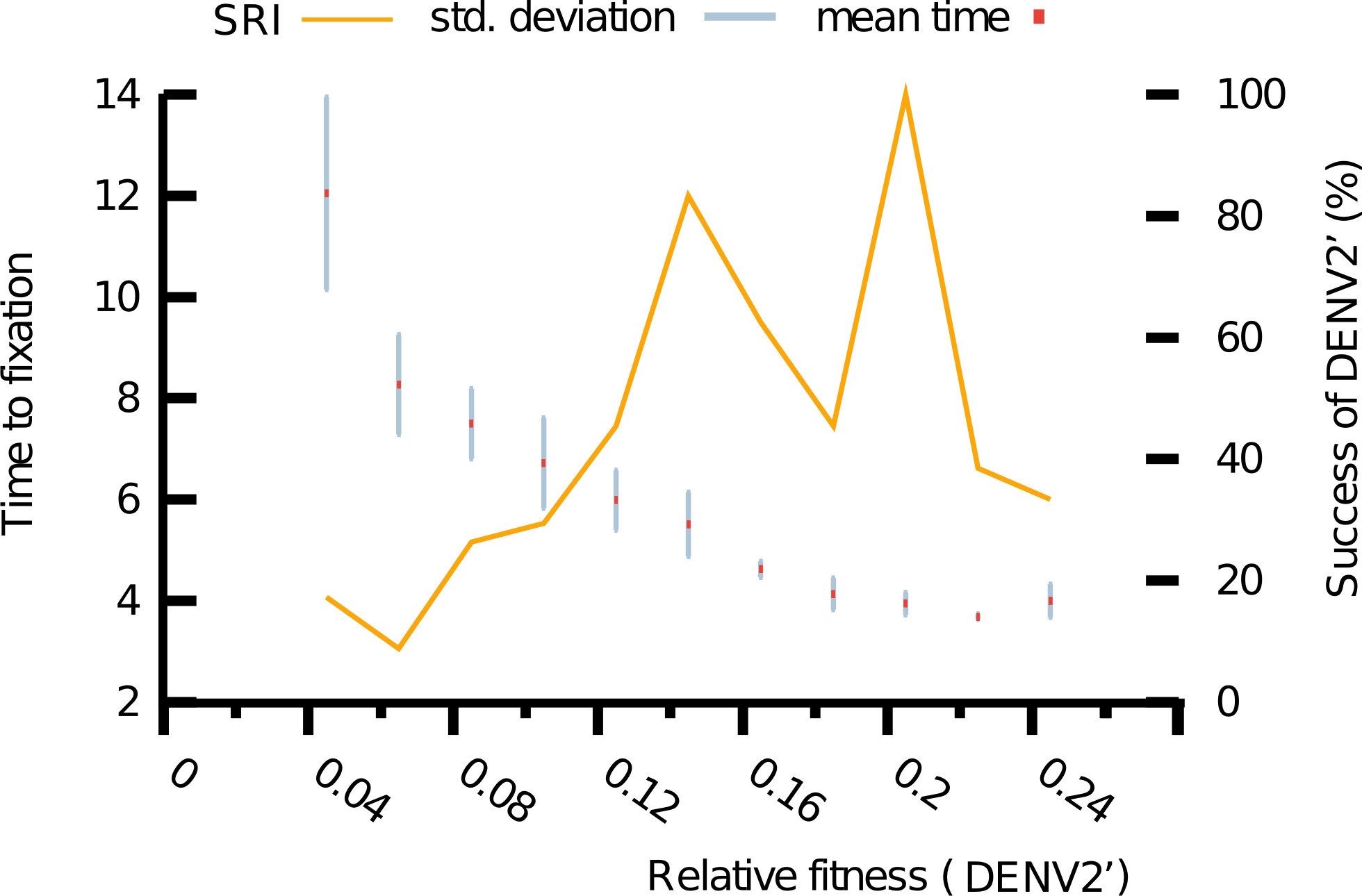

Supplement: Figure S8 — Effect of fitness advantage on invasion success. Considering a fixed time point for introduction, increasing values of ρβ result in higher invasion success rates of DENV2′ and lowers fixation time. Time of introduction 1259.5, parameter values as in Table 1 (main text). (0.29 MB TIF) [file pntd.0000894.s008.tif]

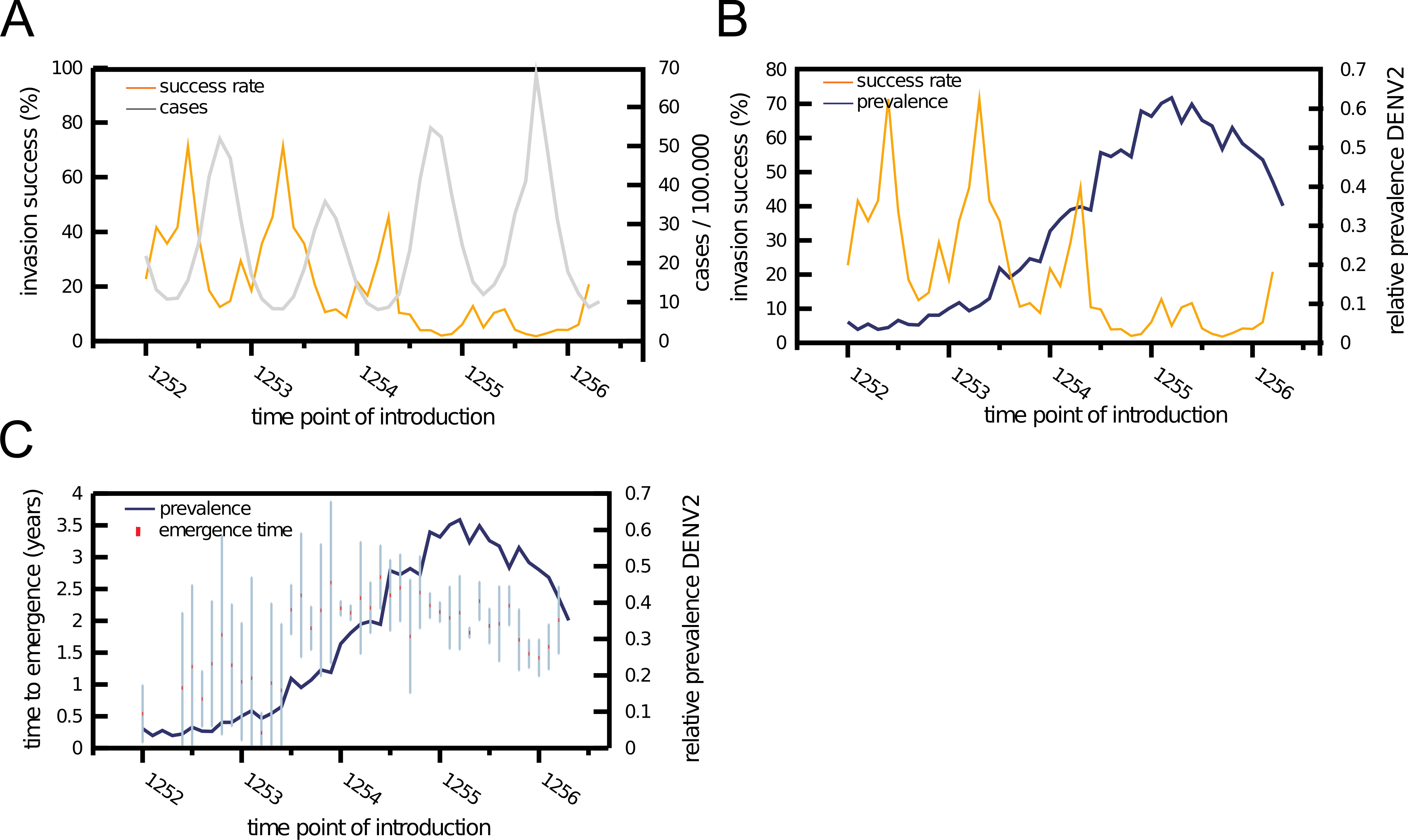

Supplement: Figure S9 — The effect of transmission and serotype competition on invasion success and emergence time of successful fixation events, assuming changes in the EIP. The success rate of the invading genotype, DENV2′, strongly varies depending on the number of total infected individuals and the relative prevalence of serotype 2 in the population at the time point of introduction (TPI). The total time required for a novel (and eventually successful) genotype DENV2′ to reach detection level is highly dependent on the relative prevalence of serotype 2 at the time it enters the population. (A) The invasion success (orange line) oscillates out of phase with total dengue incidence (grey line) and is minimized when disease prevalence peaks, demonstrating how the current level of transmission can influence the invasion success of new advantageous genotypes. (B) The highest rates of successful invasions can be observed during periods of low relative prevalence of serotype 2 (blue line). In contrast, the probability of an invading advantageous genotype to get established and reach fixation is significantly reduced as serotype 2 gains wide-spread dominant within the population. (C) The red points show how the average emergence times, i.e. the period between introduction and reaching a 10% detection threshold, of successful invasion events increases with the relative prevalence of DENV2 at the time of introduction (blue line). Standard deviations, based on 10 simulated successful invasion events, are shown as light-blue bars. Parameters as in Table 1 and ρμ = 0.045 for S9. (1.22 MB TIF) [file pntd.0000894.s009.tif]

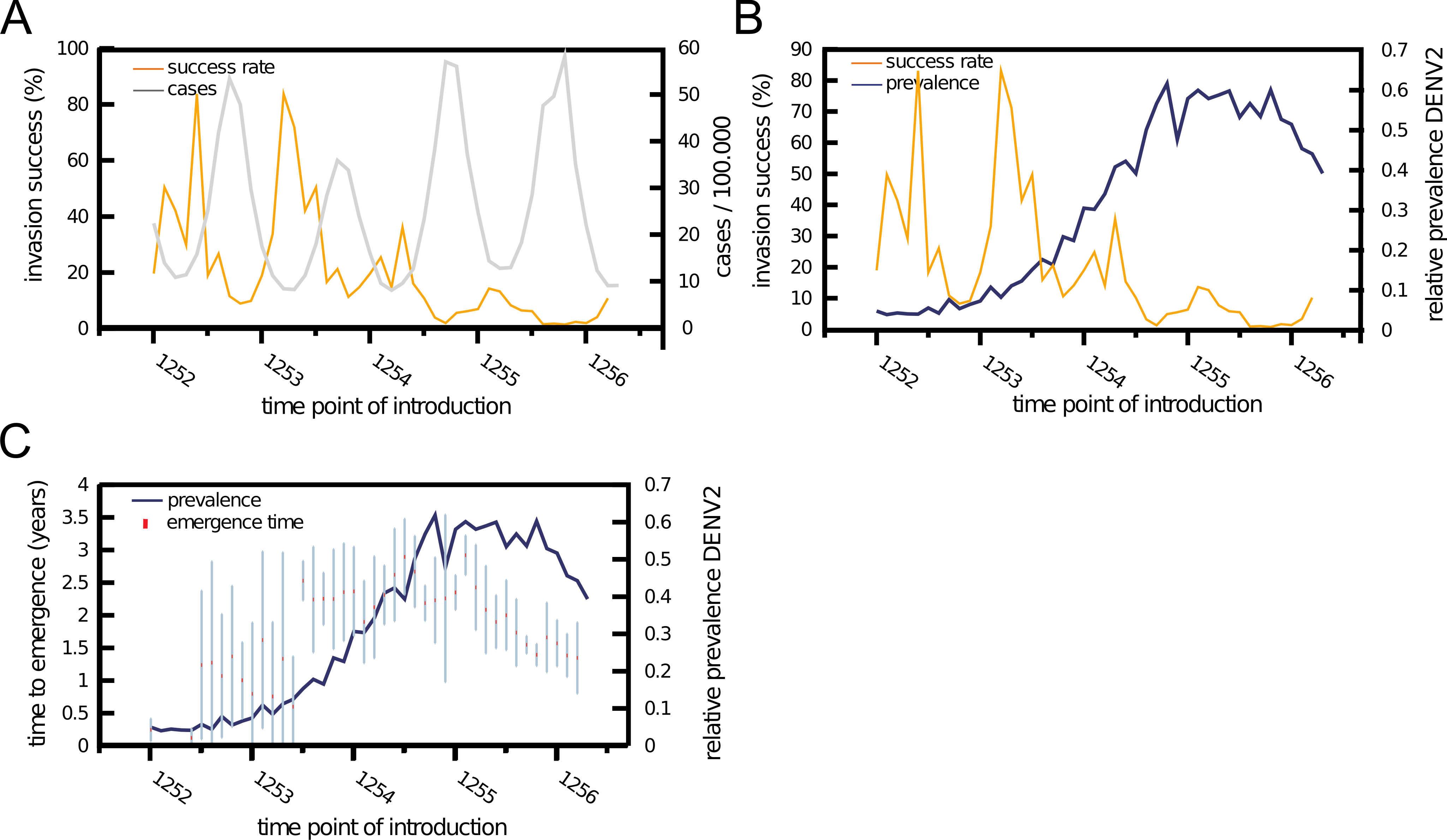

Supplement: Figure S10 — The effect of transmission and serotype competition on invasion success and emergence time of successful fixation events, assuming changes in human infectious period. The success rate of the invading genotype, DENV2′, strongly varies depending on the number of total infected individuals and the relative prevalence of serotype 2 in the population at the time point of introduction (TPI). The total time required for a novel (and eventually successful) genotype DENV2′ to reach detection level is highly dependent on the relative prevalence of serotype 2 at the time it enters the population. (A) The invasion success (orange line) oscillates out of phase with total dengue incidence (grey line) and is minimized when disease prevalence peaks, demonstrating how the current level of transmission can influence the invasion success of new advantageous genotypes. (B) The highest rates of successful invasions can be observed during periods of low relative prevalence of serotype 2 (blue line). In contrast, the probability of an invading advantageous genotype to get established and reach fixation is significantly reduced as serotype 2 gains wide-spread dominant within the population. (C) The red points show how the average emergence times, i.e. the period between introduction and reaching a 10% detection threshold, of successful invasion events increases with the relative prevalence of DENV2 at the time of introduction (blue line). Standard deviations, based on 10 simulated successful invasion events, are shown as light-blue bars. Parameters as in Table 1 and ρσ = 0.045. (1.23 MB TIF) [file pntd.0000894.s010.tif]

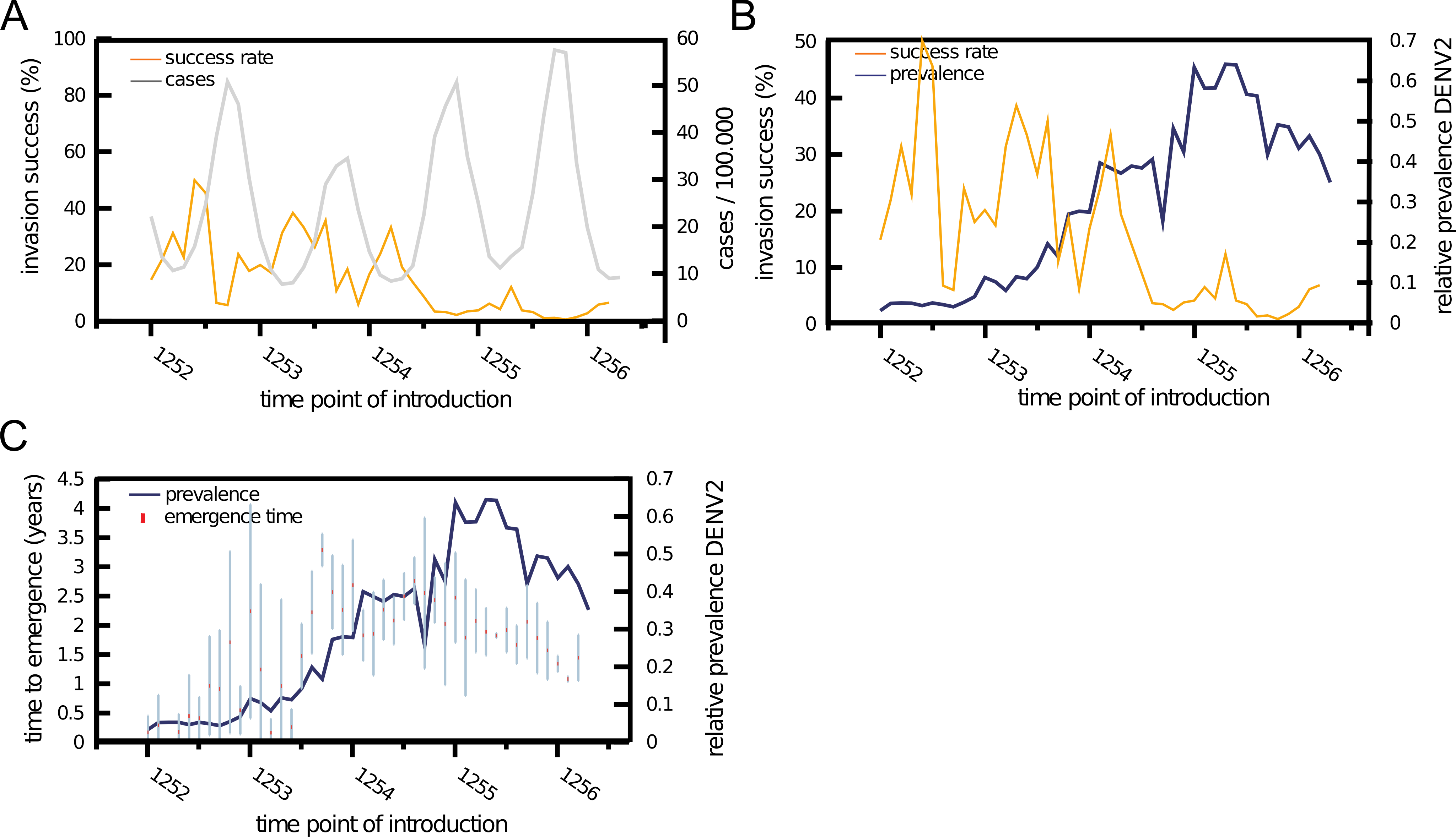

Supplement: Figure S11 — The effect of transmission and serotype competition on invasion success and emergence time of successful fixation events, assuming changes in transmissibility of secondary infections. The success rate of the invading genotype, DENV2′, strongly varies depending on the number of total infected individuals and the relative prevalence of serotype 2 in the population at the time point of introduction (TPI). The total time required for a novel (and eventually successful) genotype DENV2′ to reach detection level is highly dependent on the relative prevalence of serotype 2 at the time it enters the population. (A) The invasion success (orange line) oscillates out of phase with total dengue incidence (grey line) and is minimized when disease prevalence peaks, demonstrating how the current level of transmission can influence the invasion success of new advantageous genotypes. (B) The highest rates of successful invasions can be observed during periods of low relative prevalence of serotype 2 (blue line). In contrast, the probability of an invading advantageous genotype to get established and reach fixation is significantly reduced as serotype 2 gains wide-spread dominant within the population. (C) The red points show how the average emergence times, i.e. the period between introduction and reaching a 10% detection threshold, of successful invasion events increases with the relative prevalence of DENV2 at the time of introduction (blue line). Standard deviations, based on 10 simulated successful invasion events, are shown as light-blue bars. Parameters as in Table 1 and ρΦ = 0.075. (1.20 MB TIF) [file pntd.0000894.s011.tif]
